# Nanoscale imaging of phonon dynamics by electron microscopy

**DOI:** 10.1038/s41586-022-04736-8

**Published:** 2022-06-08

**Authors:** Chaitanya A. Gadre, Xingxu Yan, Qichen Song, Jie Li, Lei Gu, Huaixun Huyan, Toshihiro Aoki, Sheng-Wei Lee, Gang Chen, Ruqian Wu, Xiaoqing Pan

**Affiliations:** 1 Department of Physics and Astronomy, University of California Irvine, Irvine, CA USA; 2 Department of Materials Science and Engineering, University of California Irvine, Irvine, CA USA; 3 Irvine Materials Research Institute, University of California Irvine, Irvine, CA USA; 4 Department of Mechanical Engineering, Massachusetts Institute of Technology, Cambridge, MA USA; 5 Institute of Materials Science and Engineering, National Central University, Taoyuan, Taiwan

**Keywords:** Transmission electron microscopy, Quantum dots, Surfaces, interfaces and thin films

## Abstract

Spatially resolved vibrational mapping of nanostructures is indispensable to the development and understanding of thermal nanodevices^1^, modulation of thermal transport^2^ and novel nanostructured thermoelectric materials^3–5^. Through the engineering of complex structures, such as alloys, nanostructures and superlattice interfaces, one can significantly alter the propagation of phonons and suppress material thermal conductivity while maintaining electrical conductivity^2^. There have been no correlative experiments that spatially track the modulation of phonon properties in and around nanostructures due to spatial resolution limitations of conventional optical phonon detection techniques. Here we demonstrate two-dimensional spatial mapping of phonons in a single silicon–germanium (SiGe) quantum dot (QD) using monochromated electron energy loss spectroscopy in the transmission electron microscope. Tracking the variation of the Si optical mode in and around the QD, we observe the nanoscale modification of the composition-induced red shift. We observe non-equilibrium phonons that only exist near the interface and, furthermore, develop a novel technique to differentially map phonon momenta, providing direct evidence that the interplay between diffuse and specular reflection largely depends on the detailed atomistic structure: a major advancement in the field. Our work unveils the non-equilibrium phonon dynamics at nanoscale interfaces and can be used to study actual nanodevices and aid in the understanding of heat dissipation near nanoscale hotspots, which is crucial for future high-performance nanoelectronics.

## Abstract (web summary)

A method for mapping phonon momenta reveals non-equilibrium phonon dynamics at nanoscale interfaces enabling study of actual nanodevices and aiding understanding of heat dissipation near nanoscale hotspots.

## Main

The control of phonon propagation and thermal conductivity of materials by nanoscale structural engineering is exceedingly important for the development and improvement of nanotransistors, thermal barriers, phase-change memory and thermoelectric energy conversion^1^. For example, it has been a central issue to reduce the lattice thermal conductivity of thermoelectric materials for the enhancement of their figure of merit (*zT*)^2,4–7^. Although many single-phase/crystal materials already possess high *zT* values, due to their relatively high carrier mobility, their thermoelectric performance can be further enhanced by reducing thermal conductivity^3,8,9^. Numerous strategies have been used to reduce the thermal conductivity or phonon transport of a given material by introducing material intermixing^10^, nanostructures^2,4,6,9^ and interfaces^4,11^. Through these mechanisms, short-, medium- and long-wavelength phonons are scattered, respectively. The SiGe quantum dot (QD) superlattice structure is one such system, which efficiently reduces thermal conductivity by 20 times by implementing all three phonon-scattering mechanisms^12^.

Of these scattering mechanisms, interfacial phonon scattering is a subject of intense study and has been largely carried out via modelling and simulation, combined with experimental measurement of the effective thermal conductivity of many layers^13–15^, rather than by direct imaging of phonons. Although it is understood that phonon reflection at an interface is responsible for thermal boundary resistance^16^, there is no direct experimental observation of local phonon reflection. Raman spectroscopy has been used to study strain and compositional effects on phonons in SiGe superlattices^17,18^, whereas time-domain thermoreflectance measurements have been carried out to investigate thermal conductivity via ballistic transport^19^. However, both techniques lack the spatial and momentum resolution needed to study phonon dynamics of individual nanostructures and interfaces. Therefore, an experimental technique that probes nanoscale vibrational properties with high spatial, momentum and energy resolutions is vital for deepening our understanding of nanoscale phonon transport physics.

**Extended Data Fig. 1:**
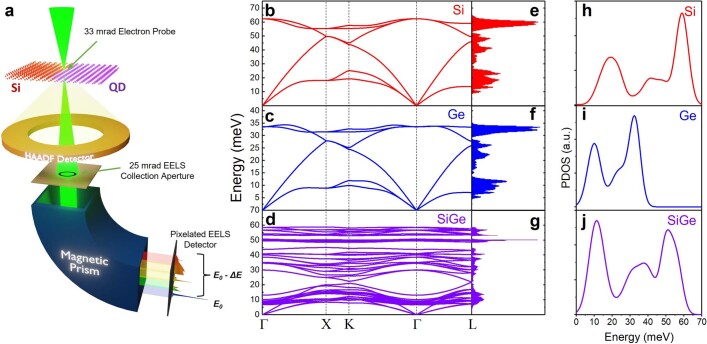
Experimental vibrational EELS set-up and phonon dispersion curves. **a**, Schematic of the STEM EELS experimental set-up. An electron beam with a convergence semi-angle of *α* = 33 mrad is focused to form a 1.5 Å wide probe on the sample. Electrons are then elastically and inelastically scattered by the sample. High-angle scattered electrons are recorded by the HAADF detector. The lower-angle scattered electrons enter the spectrometer with a collection semi-angle of *β* = 25 mrad and are dispersed based on energy. Elastically scattering electrons form the ZLP while inelastic electrons contribute to the vibrational signal. **b–d**, Phonon dispersion relations of bulk Si, Ge, and disordered Si_0.5_Ge_0.5_ respectively. **e–g**, Total phonon density of states of bulk Si, Ge, and Si_0.5_Ge_0.5_ respectively. **h–j**, Calculated phonon density of states of Si, Ge and Si_0.5_Ge_0.5_ QD respectively after convolution with a Gaussian of width 7meV.

Recent advances in monochromated electron microscopy have enabled the spectroscopy of vibrational excitations at the nanometre^20^ and even atomic^21,22^ scales. So far, two-dimensional (2D) mapping of surface and bulk excitations^23^ and detection of single-atom^24^ and defect^25^ vibrational signals have been achieved. Although dipole scattering in polar materials, such as BN^21,26,27^, MgO^23^ and SiC^20,28^, under on-axis scanning transmission electron microscopy-electron energy loss spectroscopy (STEM-EELS) the geometry produces long-range and non-local polariton modes, reducing the atomic-scale contrast in vibrational EELS signal mapping^29^, dipole scattered signals are substantially suppressed and negligible in elemental and non-polar materials with weak dipoles, such as Si^22^ and SiGe, which only contain highly localized phonon scattering. Here, we report quantitative high spatial resolution mapping of phonons in SiGe QDs using an on-axis beam-detector geometry (Extended Data Fig. 1a). We experimentally reveal a remarkable phonon signal enhancement near the interface between Si and SiGe QDs, which is confirmed to arise from the nanoscale phonon reflection near interfaces. Probing local vibrations and phonon dispersions in nanostructured semiconductors informs structure–property correlations and offers insight into the design and optimization of novel thermoelectric materials. This work serves as the foundation for future studies in nanoscale characterization of phonon propagation for the development and improvement of nanoscale structures and devices.

**Fig. 1:**
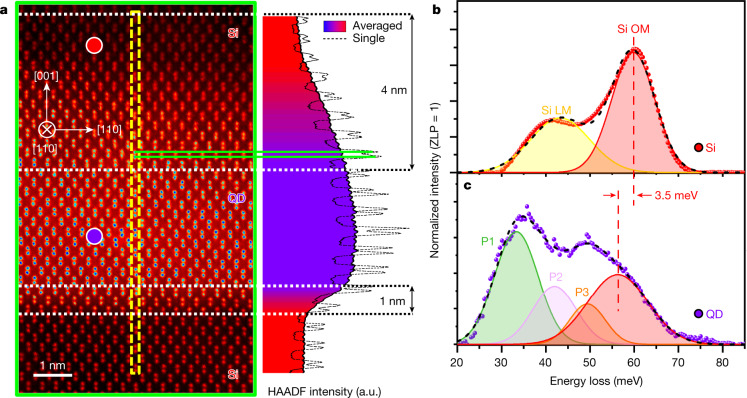
Atomic structure and vibrational spectra of SiGe QD and Si–SiGe interfaces. **a**, Atomic-resolution high-angle annular dark-field (HAADF) image containing both top and bottom QD interfaces from the region outlined in green in Extended Data Fig. 2. The line profile of a single array of atomic columns (right) is overlaid with a horizontally averaged profile of the entire image showing a gradual interface 4 nm wide and an abrupt one 1 nm wide for the top and bottom QD interfaces, respectively, estimated by the 10–90% criterion. The label for [001] denotes the growth direction and is perpendicular to the interfaces, [1$\bar{1}$0] denotes a direction that is parallel to the interfaces and [110] denotes the beam direction, which points into the page. a.u., arbitrary units. **b**, **c**, Background-subtracted, pseudo-Voigt peak separated vibrational spectra of interlayer Si and SiGe QD from locations denoted by the red and blue dots in **a**, respectively. The low energy mode (LM) represents a combination of Si LA and LO modes, whereas OM represents a combination of Si TO and LO modes. Due to the complex band structure inside the QD, we label the first three peaks shown here as P1, P2 and P3, with the 4th peak labelled as Si OM.

**Extended Data Fig. 2:**
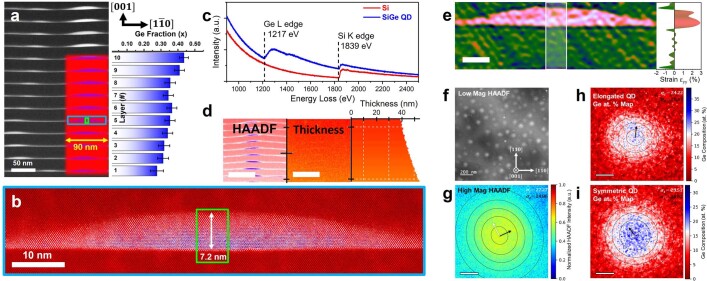
STEM characterization of quantum dot layers. **a**, Low-magnification STEM image of SiGe QD superlattice. Due to the Z-contrast property of HAADF imaging, the bright regions are SiGe, while the darker regions are interlayer Si. The crystal orientations are indicated as [$001$] for the growth direction and $[1\bar{1}0$] for the lateral, planar direction. A HAADF image of the first 10 layers from the Si substrate is superimposed with an elemental EELS map. Red and blue indicate silicon and germanium concentration, respectively. Histogram plot to the right displays Ge composition fraction of the corresponding 10 layers. **b**, HAADF image of a single QD from the blue region in **a** showing dome-like structure and dislocation-free interfaces. The green box denotes the region in Fig. 1a. **c**, Core-loss EEL spectra of interlayer Si and SiGe QD. **d**, Low-magnification HAADF image with associated thickness mapping and horizontally averaged thickness profile. QDs studied in this work were chosen in the 40–50 nm thickness range. Scale bar is 100 nm. **e**, Strain mapping of QD in **b** and interlayer Si using geometrical phase analysis (GPA) with associated line profile of the horizontally integrated region marked by the white box. Reference strain of 0% was chosen to be that of interlayer Si. Scale bar is 10 nm. **f**, Low-magnification HAADF image of several planar-view QDs with the associated crystal directions. **g**, High magnification HAADF image of a single QD showing a slight elongation of 8.8%. **h, i**, High resolution EELS Ge composition maps showing 15.5% (h) and 2.68% (i) elongation, respectively. The variances in the lateral direction were obtained via 2D Gaussian fitting. The white and black arrows denote long and short axes, respectively. The scale bars in **g**–**i** are 20 nm.

The QDs chosen for this study were grown using the Stranski–Krastanov growth mechanism (details in Methods) and those of similar dimensions were chosen for the study, to exclude size variation effects (Extended Data Fig. 2a). Each QD has a dome-like interface at the top and a flat interface at the bottom (Extended Data Fig. 2b). These results are similar to previous results supported by atomic force microscopy and TEM characterizations (Extended Data Fig. 2)^30,31^. The widths of the top and bottom interfaces were measured to be about 4 nm and 1 nm, respectively (Fig. 1a). The dome-like interface at the top of the QD and the flat interface at the bottom, closer to the substrate, are henceforth denoted as the gradual and abrupt interfaces, respectively. Elemental mapping results provide similar evidence of asymmetric Ge distribution inside the QDs as well, and can be explained by Si diffusion from the top into the Ge layer during growth. Ge fractional composition, obtained by analysing the core-loss EELS of Si K and Ge L edges (Extended Data Fig. 2c), varies almost monotonically with increasing layer number in the first several layers (Extended Data Fig. 2a) due to the growth conditions of the SiGe QD superlattice structure. Si undergoes thermal and strain-activated diffusion into the SiGe QDs, generating alloyed nanostructures^32^. As a result, SiGe QD layers closer to the substrate, which were grown first, experience higher amounts of Si diffusion than those that are further away. The varying composition of these QDs offers an interesting opportunity to utilize high-resolution vibrational electron microscopy to investigate alloying effects on local vibration within a single sample, which is not possible for macroscopic optical methods^18,33–35^.

**Extended Data Fig. 3:**
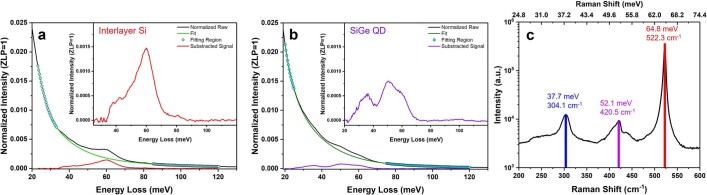
Background subtraction of low-loss EEL spectra and Raman spectrum. **a–b**, Typical background subtraction procedure of spectra acquired in interlayer Si and SiGe QD, respectively. The green coloured line represents the normalized as-acquired spectra in the corresponding regions. Cyan dots represent the spectrum region used for fitting the background. The red and purple curves in the main plot and in the inset represent the background subtracted signal respectively in **a** and **b**. **c**, Log spectrum from SiGe QD superlattice sample grown on silicon. The spectrum shows 3 distinct peaks at 37.7, 52.1, and 64.8 meV arising from Ge, SiGe, and Si optical modes, respectively. Raman spectroscopy only offers sample-averaged information of lattice vibrations.

**Extended Data Fig. 4:**
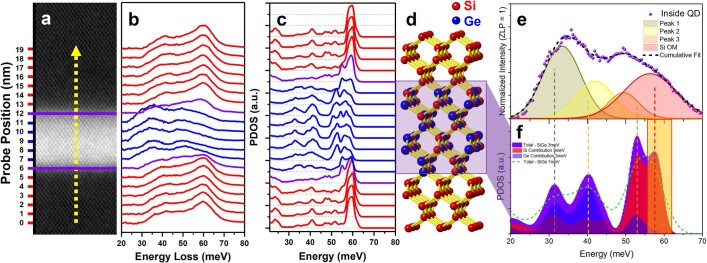
Line Scan across QD and phonon DOS of Si_0.5_Ge_0.5_ using atomistic modeling. **a**, HAADF image of the region consisting of top and bottom interfaces of a single QD. The yellow arrow notes the direction of the line scan and the purple lines mark the QD interfaces. **b**, Stacked background subtracted spectra with red, blue, and purple curves representing phonon signals in interlayer Si, SiGe QD, and interface signals. Stacked positions correspond to the probe positions labeled in **a**. **c**, Stacked calculated phonon density of states and **d**, associated atomic structure. **e**, Background subtracted, pseudo-Voigt peak separated vibrational spectra of SiGe QD. The data are replicated from Fig. 1c. **f**, 3meV-Gaussian-convolved phonon DOS of the Si_0.5_Ge_0.5_ region in **a** and its projected 3meV-Gaussian-convolved phonon DOS curves of Si and Ge. A 7meV-Gaussian-convolved phonon DOS matching the experimental energy resolution is overlaid in green. The coloured dashed lines show that positions of the prominent peaks in the phonon DOS match with the 4-peak decomposition of the experimental EELS data in **e**. The yellow box highlights the fact that the highest energy mode is almost entirely comprised of Si contribution to the modes in that energy region. The energy discrepancy between experimental data and theoretical model may arise from the different chemical composition and varied disordered structure.

To study the compositional strain inside the QD, vibrational EEL spectra were acquired using an on-axis beam-detector geometry (Extended Data Fig. 1a). In the interlayer pure Si, two distinct Si–Si vibrational peaks are visible (Fig. 1b) after spectra processing (Extended Data Fig. 3a). The peak located at 59.8 ± 0.2 meV belongs to Si transverse and longitudinal optical (TO and LO) modes, denoted as OM, and another peak to the left, which we classify as the low-energy mode signal, with an energy of 43.2 ± 0.4 meV (longitudinal acoustic (LA) and optical phonon modes near zone edges). Inside the SiGe QD, the calculated phonon density of states (DOS) in the SiGe region in Extended Data Fig. 4 suggests that there are four separable modes in the 20–80 meV range, corresponding to the various combined vibrations of the Si and Ge atoms in the SiGe alloy QD (Fig. 1c). Of these, the Si OM energy is red shifted to a value of 56.3 ± 0.3 meV due to the surrounding Ge atoms, which leads to a larger reduced mass.

There is a 5 meV discrepancy between Si OM energies from Raman (64.8 meV in Extended Data Fig. 3c) and EELS (59.8 meV) in the pure Si region. This energy offset is accounted for by noting our experimental conditions (Extended Data Fig. 1). As 33 mrad and 25 mrad convergence and collection semi-angles were used, electrons scattered at angles beyond even the second Brillouin zone (BZ) are included; a similar EELS configuration was used by K. Venkatraman et al.^22^ and is considered a momentum-averaged EELS acquisition geometry. Whereas Raman spectroscopy only probes near-zero momentum phonons at the BZ centre due to the low momentum of visible-light photons, vibrational EELS in our configuration probes phonons of all momenta, thus producing the discrepancy between our results and those in Raman literature (see detailed discussion in Supplementary Section 1). The calculated phonon densities of states (Extended Data Figs. 1f, g) match well with Fig. 1b, c, while also correctly capturing the momentum-averaged Si OM peak position. Compared to Raman spectroscopy, the outstanding advantage of vibrational EELS is the superior spatial resolution, which reveals that the vibrational signal changes abruptly to within a nanometre when the probe is moved from the interlayer Si to the SiGe QD (Extended Data Fig. 4).

**Fig. 2:**
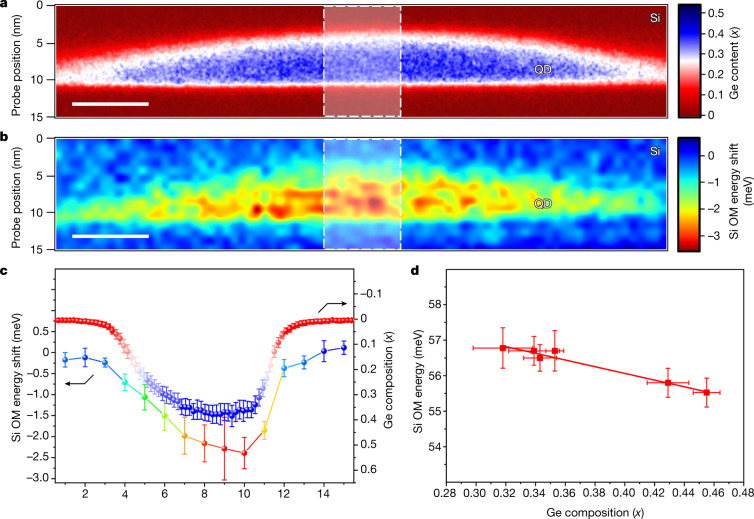
Spatial mapping of Ge concentration and Si OM energy shift in a single QD. **a**, Map of Ge composition acquired by core-loss EELS. The white contrast in the colour map makes it easy to see relatively how far the interface extends around the QD. The red and blue colours indicate nearly 0 and 50 at.% Ge concentration, respectively. **b**, Two-dimensional spatial mapping of Si OM energy shift from a nominal value of 58.9 meV of a single QD. The blue and red colour extremes denote standard and shifted energies, respectively. Scale bars in **a** and **b** are 10 nm. **c**, Ten horizontal, pixel-averaged 1D profiles (white shaded regions in **a** and **b**) of Ge composition (red-blue) and Si OM energy shift (coloured) matching the colour schemes of their respective maps. Energy shifts are obtained by subtracting 58.9 meV (average phonon energy in the surrounding Si) from all measured Si OM energy values. Error bars represent the standard deviation. **d**, Peak positions of Si OM in several QDs as germanium concentration (*x*) in the centre of the QD increases. Error bars represent the peak fitting error.

Vibrational EELS enables the nanometre correlation of elemental and vibrational information (Fig. 2a, b). The 80 nm × 15 nm dimensions of the Si OM energy-shift map cover nearly the entire QD, as well as the surrounding interlayer Si, and match well with the QD shape, confirming high spatial resolution. The Si OM energy shift is non-uniform inside the QD and has excellent tracking with the Ge composition of the QD:Si OM energy shift is highest where the Ge content is highest (Fig. 2c) with a maximum red shift of 3.88 meV. The asymmetry that is reflected in the 2D composition map is observed in the energy-shift map and consistent with the observations in Fig. 1a. Although one-dimensional (1D) nanoscale compositions of SiGe structures have been obtained^36^, we use 2D spatial composition mapping and correlate it with the composition-induced vibrational energy shift.

Furthermore, effects of varying composition across several QDs were investigated in a single sample, effectively limiting the number of free variables in our experimental set-up. Figure 2d shows the energy-shift trend of the Si OM as a function of the Ge composition (*x*) at the centre of the corresponding QDs. Our data express a linear trend with a slope of −9.3 ± 1.09 meV per *x* lying within accepted values in the literature, with values obtained by Raman spectroscopy ranging from −7.7 to −8.8 meV per *x* (refs. ^18,33–35^) and a *y* intercept of 59.8 ± 0.4 meV matching well with the momentum-averaged Si OM energy in pure Si.

**Fig. 3:**
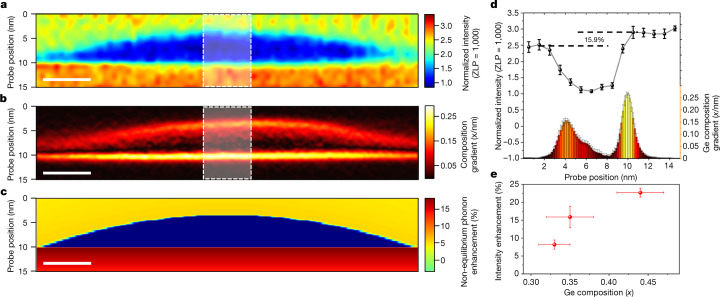
Asymmetric spectral intensities of Si OM near the gradual and abrupt interfaces. **a**, Two-dimensional spatial map of Si OM intensity of a 35 at.% average Ge composition QD. The red and blue contrast extremes denote high and low relative mode intensities. **b**, Absolute value of the vertical gradient of the elemental map in Fig. 2a, emphasizing relative interface abruptness. Scale bars in **a–c** are 10 nm. **c**, Boltzmann transport simulated mapping of non-equilibrium phonon enhancement of Si OM with top interface *SR* = 0 and bottom interface *SR* = 1. Simulation was carried out only for the Si side and only normal incident phonons are considered. Phonons with oblique incidence do not contribute to the enhancement of EELS intensity. The red colour in the map denotes a greater enhancement of non-equilibrium phonons. **d**, Ten horizontal, pixel-averaged 1D profiles of the maps in **a** and **b** (white shaded regions). The 1D intensity line profile of **a** is plotted with the histogram profile of **b**. Error bars represent standard deviation. **e**, Plot of Si OM intensity enhancement as a function of maximal Ge composition in the QD.

**Extended Data Fig. 5:**
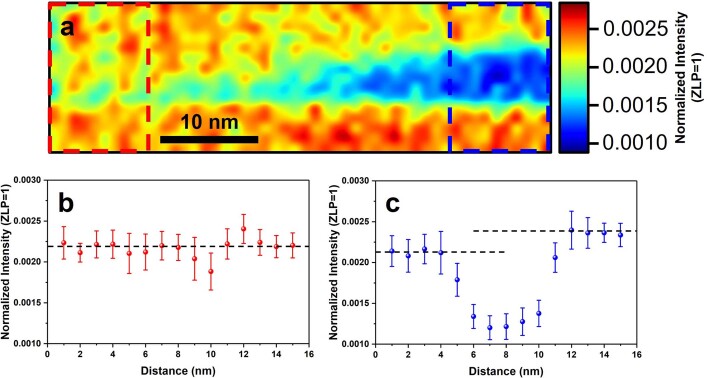
Phonon map of QD side. **a**, Spatial mapping of Si phonon peak intensity of the side of a QD. **b**, Integrated line profile of the left-hand side of the map (red) showing consistent intensity above and below the QD edge. **c**, Integrated line profile of the right-hand side of the map (blue) showing an intensity enhancement above and below the QD. The horizontal dashed lines in **b** and **c** show the intensity enhancement in the middle QD region and similar intensity in the side region. Error bars in **b** and **c** represent standard deviation.

**Extended Data Fig. 6:**
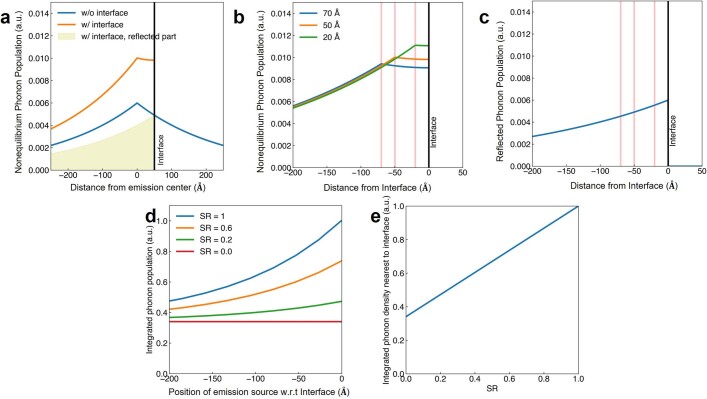
Effect of interface specularity on phonon decay. **a**, The total non-equilibrium phonon distribution function with and without the presence of an interface, and phonons that are reflected by the interface with flipped velocity direction. The blue curve describes the phonon distribution function when there is no interface. The light-yellow filled region describes the reflected phonons’ contribution to phonon distribution, which only exists in the proximity of an interface. The orange line is the phonon distribution when including the contribution of reflected phonons. The phonon emission source is placed at 0, black line indicates the location of interface. **b**, When moving the phonon emission source (marked by red bar), the phonon distribution function has a different profile. Three cases with different distances to the interface are plotted. The longer the distance from the interface, the lower the peak of the distribution, due to increasingly smaller contributions from reflected phonons. **c**, Plot of integrated area in red in plot **b** as a function of distance from the interface. The reflected phonon distribution has an exponential decay profile away from the interface and changing the beam position will probe a different amount of reflected non-equilibrium phonons. **d**, The integrated phonon population profile at various parameters of *SR*. **e**, The integrated phonon population at the position closest to the interface as a function of parameter ${SR}$.

**Extended Data Fig. 7:**
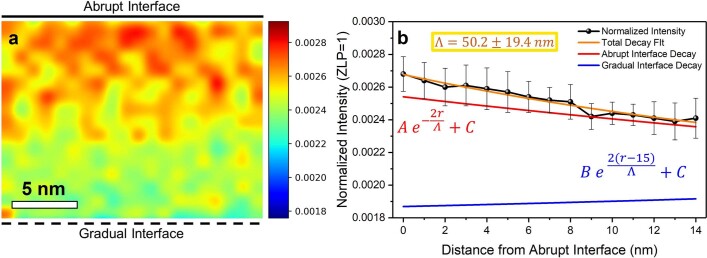
Interlayer Si Phonon map. **a**, A 20 × 15 nm map of Si phonon peak intensity in the interlayer Si region bounded by two QDs. **b**, Integrated 1D line profile of **a** with exponential decay fitting. Red and blue curves represent decay of phonon population as a function of distance from abrupt and gradual interfaces, respectively. Parameters *A* and *B* were fit to the data and represent the reflection intensity in the immediate vicinity of the interface while *C* was taken to represent the sum of the beam generated phonons *g*_0_, and equilibrium phonon population *f*_0_, that the reflected population would eventually decay to. The measured value for Si OM MFP is 50.2 ± 19.4 nm where the uncertainty of each point is factored into the total error. The spot-like features in **a**, which also appears in Fig. 3a, may arise from on-column/off-column effect. A lateral periodicity of 3.33 nm measured from fast Fourier transform (FFT) of **a** is much larger than the (1$\bar{1}$0) interplanar distance (0.384 nm) due to the sampling interference between atomic lattice and 1-nm step size. However, the rigorous correlation with atomic position needs careful consideration of experimental conditions such as the probe defocus, sample thickness and EELS collection angle, because the contrast of on-axis vibrational loss signals acquired on an atomic column and between columns can be reversed due to electron beam channeling^29^. Error bars represent standard deviation.

Figure 3a shows a 2D intensity mapping of the Si OM obtained from the same QD as in Fig. 2b. A striking feature is that the Si OM at the bottom interlayer Si has a 15.9% intensity enhancement relative to that of the top one, highlighted in Fig. 3d, despite there being no compositional variance in the interlayer Si, as evidenced by Fig. 2a. We posit that the source of this enhancement arises from the distinct scattering dynamics due to the two differing interfaces: an abrupt bottom interface and a gradual top interface (Fig. 3c, d). The momentum-averaged scattering cross-section is given by *σ* = ∫*σ*(*ω,****q***)d***q*** and the momentum-resolved Stokes scattering probability of fast electrons (see ‘DPM mapping’ for details and definitions of terms) due to the lattice vibration near an interface has been derived^26^. To explain the experimental intensity enhancement, the local variations of equilibrium phonon population *n*_***q****,v*_, where ***q*** denotes the phonon momentum and *ν* denotes a certain phonon branch, and local density of states (LDOS) were considered individually. As the beam-induced temperature rise is negligible (see Supplementary Section 4), *n*_***q****,v*_ is constant in two Si regions near the abrupt and gradual interfaces. The computed LDOS showed no intensity enhancement, which was probably due to the unchanged composition (Supplementary Fig. 1). Hence, the local non-equilibrium phonon occupation *f*_***q****ν*_ is determined to be a dominant factor for producing the intensity enhancement below the abrupt interface in Fig. 3a (see Supplementary Section 6). The non-equilibrium phonon occupation consists of two parts *f*_***q****,ν* _*=* *f*_***q****,ν*,0 _+ *g*_***q****,ν*_, with *f*_***q****,ν*,0_ being the equilibrium Bose–Einstein distribution (*n*_***q****,v*_) at room temperature and *g*_***q****ν*_ being the population deviation from equilibrium due to electron energy loss and interface reflections. Near the interfaces, optical phonons in the Si side are reflected by the interface due to mode mismatch. Thus, the population deviation *g*_***q****,ν*_ can be further decomposed into two parts, as the phonons emanating from the electron beam, *g*_0_, and the specularly reflected phonons from the interface. We use the Boltzmann transport equation (BTE) to solve for population deviation *g* and find that *g* = *g*_0_ +  *DSR*exp(−2*b*/*Λ*), where *D* is a prefactor, *S* and *R* are the specularity parameter and reflectance of the interface, respectively, *b* is the distance to the interface and *Λ* is the phonon mean free path (MFP). From atomistic Green’s function calculation, we find a minimal difference in the reflectance for an abrupt interface and a gradual interface as a function of nanostructure curvature (Supplementary Fig. 5). Thus, we conclude that the difference in the non-equilibrium phonon population must be attributed to the difference in *S* of the abrupt interface and the gradual interface. Our BTE simulation shows that the EELS intensity enhancement is attributed to higher *S* of the abrupt interface than the gradual interface. As the Ge content in the QD is increased, the composition change at the abrupt interface becomes more drastic, leading to an intensity enhancement that exhibits a monotonic relationship with increasing Ge composition inside the QD, as shown in Fig. 3e. Furthermore, the enhancement fades as phonons at the end of the QD are probed, where the gradual and abrupt interfaces become nearly identical (Extended Data Fig. 5), because the thinner interface may have a smaller reflectance, thus reducing the reflected phonon population equally on either side of the interface. Additionally, phonon reflection can be used to study phonon MFP (Extended Data Figs. 6 and 7).

**Extended Data Fig. 8:**
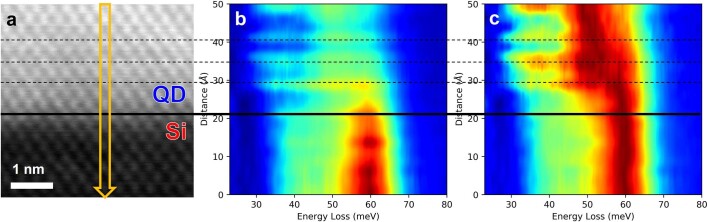
Atomically resolved line scan across bottom abrupt QD interface. **a**, Atomic resolution HAADF image of an abrupt QD interface. The downward yellow arrow indicates the direction of the phonon line scan. Solid lines mark the atomic positions in the HAADF image while the dashed line marks the Si–QD interface. **b**, Contour plot of line scan spectra normalized to the ZLP. Fringes are clearly seen and correlate with the atomic positions in the HAADF image in **a**. **c**, Spectral contour plot normalized to the signal maximum. Fringes are only seen on the Ge optical mode while the intensity on the Si optical mode is uniform. Such features could presumably correlate with the formation of sub-Ångström resolution HAADF STEM images, in which there is a signification contribution from localized phonon-scattering to the image contrast^45^. This relationship implies that sub-Ångström resolution could be achievable in the vibrational EELS.

The variation of vibrational signal in the line scan (Extended Data Fig. 8b) presumably arises from the short-range coulomb interaction between the beam and the atomic nucleus, providing atomic-scale contrast^29^. Interestingly, even when the maximum peak heights are normalized to 1, the Ge OM still shows a strong modulation.

**Fig. 4:**
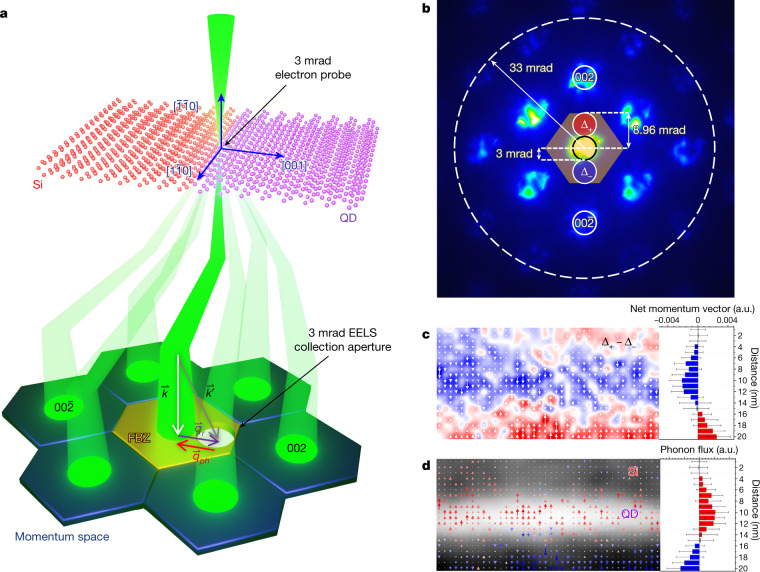
Momentum-resolved DPM map of phonon momentum normal to gradual and abrupt QD interfaces. **a**, Schematic of the experimental beam geometry showing an off-axis position of the EELS aperture in the first Brillion Zone (FBZ) of Si, achieved via post-specimen tilting of the scattered electron beam. The crystal axes are superimposed on the Si–QD crystal structure, and the momentum conservation vectors of the electron–phonon energy exchange process are superimposed on the momentum space portion of the diagram. The electron scattering wavevector diagram illustrates the fast electron–phonon momentum transfer. Under these momentum-resolved experimental conditions, ***q*** ≈ ***q***_***r***_ and since the generated phonons have an opposite sign to the momentum change of the electron beam, the momentum of generated phonons is predominantly in the in-plane direction (perpendicular to the interface). **b**, The 3 mrad convergence semi-angle convergent beam electron diffraction pattern where the 002 and 00$\bar{2}$ spots are labelled. The slightly transparent orange hexagonal shape in the centre denotes the FBZ of Si with the Γ–X distance labelled as 8.96 mrad. The red and blue circles near the centre denote the EELS entrance aperture positions where off-axis data were acquired. **c**, Mapping of net vertical momenta of Si optical phonons. **d**, Phonon flux vector map obtained from the product of group velocity and the quantity of phonons with a given momentum direction overlaid on a 3 mrad HAADF image. Panels **c** and **d** are accompanied by their respective horizontally averaged line profiles. Error bars in **c** and **d** represent standard deviation.

To recover directional and momentum information and elucidate phonon dynamics at the QD interfaces, a momentum-resolved beam-detector geometry was used with a 3 mrad convergence semi-angle^25,37^ (Fig. 4a) to obtain a differential phonon momentum (DPM) map (see ‘DPM mapping’). Mapping the difference and considering momentum conservation (the phonon momentum vector is opposite to that of the electron momentum change direction, as shown in Fig. 4a), a DPM map in the vertical direction is generated and phonon momenta directions are experimentally imaged at the nanoscale for the first time. For an atomically ideal interface, phonon reflection at interfaces is considered to be specular where the momentum parallel to the interface ***q***_**//**_ is conserved due to the translational symmetry as seen. Conversely, for an atomically irregular interface, the atomic disorder breaks the transverse translational symmetry and, consequently, phonon modes with different ***q***_**//**_ can also be coupled, leading to a diffuse scattering process. The DPM map in Fig. 4c shows momentum vectors pointing towards the abrupt interface and, given that optical phonons in Si have a group velocity in the opposite direction of their momentum from Γ–X (Extended Data Fig. 1), Si optical phonons are then taken to be propagating away from the abrupt interface. The phonon flux, the product of group velocity and quantity of phonons with a given momentum shown in Fig. 4d, experimentally confirms this phenomenon and is consistent with the physics encapsulated by Fig. 3a, b. The more abrupt interface has a higher degree of specularity and therefore the generation of backward-moving phonons is preferred, whereas the more gradual interface at the top has much weaker directional preference due to its more gradual transition from Si to SiGe.

The ability to spatially map vibrations in nanostructured semiconductors is of paramount importance in the field of energy conversion, quantum computing and nanotechnology in general. We have demonstrated that vibrational EELS is capable of probing interaction dynamics that manifest as modulations in the local population of vibrational states. Our results offer insight into nanoscale phonon physics at interfaces and show experimentally modulations in local vibrational states in the presence of nanostructures and interfaces. We have also obtained direct experimental evidence of dynamic phonon processes in the form of phonon reflection from interfaces at the nanoscale, unveiling the interplay between phonons and interface specularity. In conclusion, it is optimal for nanostructures to have an abrupt change in structure to achieve high phonon impedance thus lowering thermal conductivity. Beyond thermal transport, combining subnanometre resolution with vibrational information offers an unprecedented level of access into nanoscale thermodynamics, such as heat capacity and entropy.

## Methods

### Fabrication of SiGe QDs and preparation of TEM sample

The QD sample was grown at 600 °C in an ultra-high vacuum chemical vapour deposition (UHV/CVD) system. For Ge and Si depositions, pure GeH_4_ and SiH_4_ gases were used as precursors, respectively. The Si wafers were first etched in a diluted HF solution to create a hydrogen-passivated surface, before deposition. After deposition of a 50 nm thick buffer layer of Si, Ge layers were grown with 20 nm Si spacer layers in between each Ge QD layer for the formation of self-assembled conventional QDs. For investigating thermal stability and tuneability of the structural parameters, in situ post-deposition annealing was conducted at the growth temperature for 1 h. The final product was a thin-film-like material of 40-period multifold QD stacks with a thickness as high as approximately 1.2 µm. QD nanostructures spanned 70–90 nm across and were 6–8 nm thick (see Extended Data Fig. 2). The SiGe QDs have a diamond-like structure similar to that of Si and Ge, with a random and disordered arrangement of Si and Ge atoms. More details of the growth process can be found in ref. ^30^. The cross-sectional TEM sample used in this study was prepared by focused-ion-beam milling, whereas the planar-view TEM sample was prepared by mechanical polishing. The QD interfaces were normal to the (001) crystallographic plane, whereas the zone axis was along the [110] crystallographic direction. Of all the QDs investigated, several were elongated by up to 10–15% (Extended Data Fig. 2g, h) in no particularly consistent direction. However, there were some quite symmetric SiGe QDs (Extended Data Fig. 2i).

### Raman spectroscopy

Raman spectra were acquired using a Renishaw inVia confocal Raman microscope. Point spectra in the QD sample and Si wafer were acquired in 50 one second frames and then summed to achieve high signal-to-noise ratio.

### STEM imaging

Images were taken with the Nion UltraSTEM 200 high-energy resolution monochromated EELS system (HERMES) operating at 60 kV and 200 kV acceleration voltages with 33 mrad and 34 mrad convergence semi-angles, producing 1.5 Å and 0.78 Å sized probes, respectively. The high-angle annular dark-field signals were collected using a high-angle annular detector with inner and outer collection angles of 103 mrad and 210 mrad, respectively. Beam current was approximately 100 pA in the imaging condition. The sizes of the QD interfaces were estimated using the 10–90% criterion^38^.

### Composition EELS mapping

2D planar and cross-sectional elemental maps were acquired using the double Cs-corrected 300 kV JEOL Grand ARM S/TEM system. A 0.5 eV per pixel dispersion was used for elemental mapping, with a convergence semi-angle of 25 mrad and a collection angle of 100 mrad. Spectra were acquired in the 500–2,355 eV range, including Ge L edge at 1,217 eV and Si K edge at 1,839 eV (Extended Data Fig. 2c). The core-loss spectrum in the interlayer Si shows only one peak, corresponding to the Si K edge, whereas the spectrum in the SiGe QD shows two peaks, corresponding to the Ge L edge and Si K edge. Single QD elemental maps were acquired with a 0.5 nm per pixel step size, whereas low-magnification spectra were acquired with a 2 nm step size and were used to determine the composition distribution in different QD layers. The core-loss signals of Si and Ge were background subtracted and integrated using Gatan’s Digital Micrograph software. The histogram data reported in Extended Data Fig. 2a were obtained by averaging 4 × 4 pixel centres of each QD.

### HERMES experiments

Experimental data were acquired on a Nion UltraSTEM 200 microscope with the HERMES system operating at 60 kV, to achieve a balance between high spatial resolution and high inelastic scattering probabilities by electrons for vibrational spectroscopy. EEL spectra were produced by low-angle scattered electrons (Extended Data Fig. 1a). Combining the monochromator with a high-dispersion spectrometer, we achieved the best energy resolution of 5.7 meV with the probe in vacuum for a 10 ms acquisition at 60 keV. A CMOS camera collected a 2D spectrum image, which was then cropped and flattened in the undispersed direction to produce a 1D EELS spectrum.

### Momentum-averaged 33 mrad condition

EELS acquisition was performed using a 33 mrad high-current probe with resolution and beam current as described in the section ‘STEM imaging’. A high probe current is necessary for attaining high signal-to-noise ratio spectra, at the cost of slightly decreasing the spatial resolution. Insertion of the monochromating slit reduces the probe current to about 3 pA, but gives an energy width of 8.3 meV for an acquisition time of 1 second with the probe placed on the sample. This enables accurate probing of the optical modes of germanium. An EELS entrance aperture of 1 mm subtends a 25 mrad EELS collection semi-angle. With the BZ of silicon having a Γ–X length of only 8.96 mrad, a collection semi-angle of 25 mrad collects inelastically scattered electrons from multiple neighbouring BZs as well.

Spectral data were acquired in the form of line scans and 2D maps with the monochromating slit inserted. For line scans, 100 spectral frames were collected at 1 second exposure, aligned by their respective zero-loss peak (ZLP) maximums and then summed for each probe position. The dark current spectrum was frequently obtained during experiments. Aligning individual frames by ZLP averages for any random noise present in each frame. Maximizing single-exposure time without saturating the EELS camera provided the best signal-to-noise ratio. Implementation of this acquisition scheme was achieved using Nion Swift software and custom Python scripts designed to directly control the necessary hardware parameters.

Phonon maps of an entire SiGe QD consist of nanometre-step 80 × 15 arrays of data points that were obtained with 1–1.5 second exposure and 5 frames per pixel. Due to the large size of the map, the number of integrated frames was optimized to limit sample drift and drops in emission current overtime. A typical acquisition time for maps was about 2–3 h and presented several challenges for acquiring high quality data: sample contamination, drift, gradual drop in beam current and deterioration of energy resolution due to low-order spectrometer aberrations. To mitigate these effects, the following precautions and procedures were undertaken:

1. The instrument was tuned to optimal EELS acquisition parameters achieving an energy resolution of about 8.3 meV on the sample and was stabilized for at least 4 h. The experiment was then performed the morning after and, owing to the remarkable stability of the UltraSTEM, a sample drift of less than 5 Å per hour was present.
2. Custom mapping code enabled the flashing of the tip every hour to ensure a high beam current, which was necessary for optimal signal-to-noise ratio as well as the automatic correction of EELS first-order aberrations every few points, to maintain a high-energy resolution throughout the experiment.

### Momentum-resolved 3 mrad condition

**Extended Data Fig. 9:**
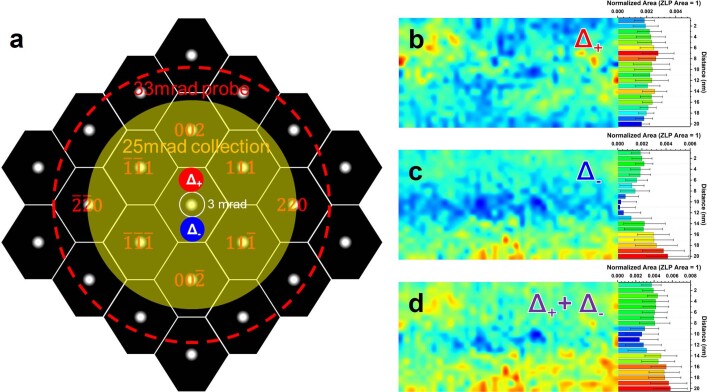
Momentum-resolved experimental beam geometry. **a**, Reciprocal space schematic diagram of Si. White borders denote individual Brillouin zones (BZ) with the BZ’s near the center labeled. White spots at the center of each BZ depict areas of dominant elastically scattered electrons while the black background is dominated by inelastically scattered electrons. Various beam-detector geometries corresponding to momentum-averaged and momentum-resolved conditions are denoted by circles with their size in semi-angle labeled. Red and blue regions in the FBZ correspond to the areas in reciprocal space where the DPM data was obtained. **b**, **c**, Mapping of integrated background subtracted spectral intensity in the 55–65 meV region acquired from electrons deflected towards the 002 (Δ_+_) and $00\bar{2}$ (Δ_−_) crystal direction corresponding to the red and blue regions in **a**, respectively. **d**, Combined spectral intensity of Si OM peaks from Δ_+_ and Δ_−_ producing a similar intensity enhancement as seen in Fig. 3a of the main text. Phonon momenta are taken to be in the opposite direction of electron deflection. Error bars in **b**–**d** represent standard deviation.

With a 3 mrad convergence semi-angle and 2.5 mrad EELS collection semi-angle, the real-space probe size was estimated to be 2.6 nm and on-axis energy resolution to be 8.3 meV in vacuum and 9.1 meV on the sample. DPM mapping was carried out by collecting off-axis spectral intensity within the first Brillion Zone. Off-axis collection was achieved by using a combination of post-specimen lenses to shift the diffraction image while keeping the real-space probe stationary, so that the region of interest in reciprocal space lies on the EELS entrance aperture (Extended Data Fig. 9). Energy resolution in this condition was estimated to be 12–14 meV, making peak separation difficult. Acquired maps were 30 × 20 in size with a step size of 1 nm per pixel and 5 s exposure. The sample was stabilized to minimize drift so that data from both areas symmetric about the Γ could be acquired from the same QD with less than 1 nm of drift.

### Spectral data processing

All acquired spectra were processed using custom Python codes. Spectra were first normalized with respect to the ZLP maximum so that each vibrational intensity represented a fractional scattering probability. Spectra were then background subtracted using a linear combination of a Lorentzian centred on the ZLP and an exponential polynomial e^*p*(*x*)^, where *p*(*x*) is an even fourth-order polynomial. This function was fitted to energy windows before and after the regions of interest. This combination produced a sharply decreasing left end and a slow and stable right tail, which were necessary for accurately extracting the low-energy optical modes of germanium. The fit was obtained using scipy.optimize, a Python library and fitting coefficients, and covariances were extracted. To determine the efficacy of the background fit, error values obtained from the square root of the diagonal terms of the covariance matrix, were examined and minimized. The background-subtracted signals were scrutinized for any negative, non-physical values.

By fitting the vibrational EELS signal with pseudo-Voight fits, which are linear combinations of Gaussians and Lorentzians, we can gather information about a mode’s excitation probability and its energy position, given by the fitting intensity and peak-position outputs. Individual inelastic probabilities were separated from background-subtracted spectra by performing pseudo-Voight fits using curve_fit() from scipy.optimize. Fitting parameters of individual peaks and their corresponding errors were extracted. We examined the value of the errors obtained from the covariance of the fit to validate our peak separation. Due to the nature of the convoluted signal, phonon dispersion curves and Raman scattering data were used to accurately deconvolute the overlapping peaks. Lower bounds of 0 and upper bounds of infinity and 40 meV were placed on the height and width of the pseudo-Voight fits, respectively. The energy position bounds were chosen to be ±5 meV from their initial reference position to avoid crossing of the separate pseudo-Voight fits.

Momentum-resolved spectra were first trimmed and processed by binned principal component analysis (PCA) before background subtraction due to the low phonon scattering cross-section under this beam geometry. After background subtraction, due to the poor energy resolution and low intensity, peak separation was not feasible, and a simple integration was carried out instead to obtain the Si optical mode intensity. The 2D maps were created by integrating the signal in the 55–65 meV energy region in each pixel.

### Principal component analysis

Given the acquisition parameters of the line scans, the excellent signal-to-noise ratio was adequate to accurately separate the individual modes. The low acquisition parameters for mapping datasets required the use of PCA to improve the signal quality. Rather than using Fourier filtering or other smoothening techniques, PCA learns from the large map dataset and reconstructs the spectra, maintaining important features while improving their quality.

**Extended Data Fig. 10:**
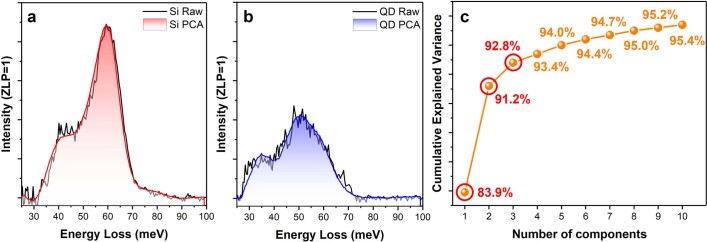
Principal Component Analysis of Background-Subtracted Spectra. **a**, **b**, Raw background subtracted spectra in Si and QD plotted with PCA reconstructed data, respectively. **c**, Scree plot of cumulative explained variance showing 10 eigenvector decomposition with  negligible explained variance after the first 3 eigenvectors. Therefore, the first 3 eigenvectors were chosen for the decomposition giving a cumulative explained variance of 92.8%. PCA reconstructed plots in **a** and **b** show that 3 eigenvectors sufficiently smoothen the data without any loss of features.

Map data were first background subtracted and trimmed to reduce feature size. The dataset was then arranged as *N* × *D*, where *N* represents background-subtracted spectra at different points and *D* the pixel intensity of each individual signal. Three principal eigenvectors described most of the data and including additional eigenvectors offered only a fraction of a percent increase in cumulative explained variance. PCA was used on map sizes of 80 × 15, producing a total of *N* = 1,200 data points and trimmed background-subtracted spectra of only *D* = 182 pixels, achieving an ideal condition for this machine-learning algorithm to produce excellent results (Extended Data Fig. 10).

Off-axis momentum-resolved data could not be smoothed the same way due to the smaller collection angle and modified phonon-scattering cross-section. In the standard implementation of PCA, not every sample is included and therefore it fails to smooth the data as the dominant feature is noise. Instead, we created a superset of the map data by binning the pixels and adding them to the original map dataset. Binning averages out the noise while enhancing the spectral features of interest. First, map data were binned by 2 then 3, etc., with each successively added to the original dataset to create a superset. With the addition of binned data back into the dataset, PCA performs as intended and accurately smooths the data. The same criteria as above were used in selecting the proper number of eigenvectors.

### Data visualization

Contour plots of mapping data were created using matplotlib.pyplot, another Python library. Extracted parameters from the pseudo-Voigt fitting were then used to create a spatially resolved 2D map. Maps were generated using a Gaussian interpolation for better visualization. Phonon-mode intensity maps and peak-position maps were constructed for silicon optical modes in units of normalized intensity and meV, respectively.

### Multimodal simulation approaches

We used three complementary computational methods (first-principles calculation, Green’s function and BTE) to simulate the non-equilibrium contributions from the beam and interface reflections. First, to understand the experimental vibrational EEL spectra from interlayer Si and SiGe QDs, first-principles calculations were used to carry out a precise simulation of phonon band structure and partial DOS (PDOS) of Si, Ge and disordered SiGe alloy (see the next section and Extended Data Figs. 1 and 4). First-principles calculations were also performed to consider the strain effect in interlayer Si (see Supplementary Section 3 and Supplementary Fig. 2). The weak tensile strain in the Si near the abrupt interface will slightly decrease the PDOS of Si OM, which cannot explain the enhancement of EELS intensity. Second, to further understand the enhancement of Si OM intensity in Fig. 3, we used Green’s function to investigate the effect of the QD geometry in a large supercell containing both gradual and abrupt interfaces (see Supplementary Section 2 and Supplementary Fig. 1), which cannot be handled by the first-principles calculations. However, the local phonon DOS results do not show a notable enhancement of PDOS near the interface, to interpret the change of vibrational signal of optical phonons in Fig. 3. The EELS cross-section is intimately related to the vibrational properties of atoms, including the phonon distribution function and phonon dispersion, whereas the non-equilibrium phonon part of the phonon population is closely related to the reflection coefficients (see equations (1) and (2) below). Finally, we narrowed down the source of the enhancement to the non-equilibrium distribution of phonons generated due to the energy transfer from fast electron to the sample. We used the BTE (see Supplementary Section 6, Fig. 3c, Supplementary Fig. 7, as well as Extended Data Fig. 6) to compute the non-equilibrium phonon population. In BTE simulations, the mode-resolved phonon reflection coefficients by the abrupt and gradual interfaces are obtained from atomistic Green’s function (see Supplementary Section 5 and Supplementary Figs. 5 and 6). The phonon Boltzmann transport describes the phonon dynamics, and we adopt the relaxation time approximation in solving the phonon BTE, which has been shown to be a good approximation in SiGe alloys^39^.

### Phonon dispersion and DOS simulations

Our first-principles calculations were carried out using the Vienna ab-initio simulation package with the projector augmented wave method. This method was adopted for the interaction between valence electrons and ionic cores^40,41^, where the energy cut-off for the plane-wave basis expansion was set to 700 eV. The generalized-gradient approximation with the functional developed by Perdew–Burke–Ernzerhof was chosen for the exchange-correlation functional^42^. All atoms were fully relaxed using the conjugated gradient method for the energy minimization until the force on each atom became smaller than 0.01 eV Å^−1^. The phonon spectrum and the corresponding phonon DOS were obtained using density functional perturbation theory^43^. To compare with the experimental phonon signals, the phonon DOS was convolved with a Gaussian of width 7 meV to match the energy resolution of the ZLP.

### DPM mapping

Well-established momentum-resolved approaches can measure phonon dispersion curves along certain reciprocal directions and reveal the local spectral variation induced by exotic phonon modes. However, such approaches cannot identify the direction of phonon propagation, which is essential for understanding the heat transport in real devices. To obtain the propagation direction of specific phonons, we developed a DMP method by subtracting phonon states at opposite reciprocal locations. Although state-of-the-art methods in momentum-resolved vibrational EELS have had great success in measuring phonon dispersion curves^26^, and studying nanoscale defect modes^25^, they have not yet taken advantage of the momentum polarization selection that becomes available in this configuration.

To recover directional and momentum information and elucidate phonon dynamics at the QD interfaces, a momentum-resolved beam-detector geometry was used (Fig. 4a) to obtain a DPM map. A convergence semi-angle of 3 mrad (momentum resolution of 0.5 Å^−1^) was used here, which was about one-third the size of the Si BZ, as shown in Fig. 4b, enabling momentum resolution, whereas the 33 mrad area encompasses even neighbouring BZs (Extended Data Fig. 9a). Integrated spectral intensity in the 55–65 meV region (Si OM) in the red and blue regions represents Si OM phonons created by electrons deflected towards the 002 (Δ_+_) and $00\bar{2}\,$ (Δ_–_) regions in reciprocal space, respectively (Extended Data Fig. 9b, c).

A theoretical description of DPM imaging begins with the EELS scattering cross-section, which takes into account the non-equilibrium phonons by replacing the equilibrium phonon occupation ${n}_{{\boldsymbol{q}},\nu }$ with the complete phonon population *f*_***q****,ν*_ = *n*_***q****,ν*_ + *g*_***q****,ν*_, where *g*_***q****,ν*_ denotes only the non-equilibrium contribution^26^:

1 $$\frac{{{\rm{d}}}^{2}\sigma ({\boldsymbol{q}},\omega )}{{\rm{d}}\Omega {\rm{d}}\omega }=\frac{4}{{{a}_{0}}^{2}}\frac{\hslash }{{q}^{4}}{\sum }_{v}\frac{{f}_{{\boldsymbol{q}},\nu }+1}{{\omega }_{{\boldsymbol{q}},\nu }}{|{\sum }_{I}\frac{1}{\sqrt{{M}_{I}}}{F}_{I}({\boldsymbol{q}}){\boldsymbol{q}}\cdot {{\boldsymbol{e}}}_{{\boldsymbol{q}},\nu }^{I}{e}^{-i{\boldsymbol{q}}\cdot {{\boldsymbol{\tau }}}_{I}}|}^{2}\delta (\omega -{\omega }_{{\boldsymbol{q}},\nu }).$$

Here, *a*_0_ is the Bohr radius, *ħ* is reduced Planck's constant, ***q*** is the momentum transfer, ${\omega }_{{\boldsymbol{q}},\nu }$ is the phonon frequency for momentum transfer ***q*** and phonon branch $\nu$, ${M}_{I}$ and ${{\boldsymbol{\tau }}}_{i}$ are the atomic mass and position, ${F}_{I}({\boldsymbol{q}})$ is the component in the Fourier transform of the charge density associated with atom $I$ and ${{\boldsymbol{e}}}_{{\boldsymbol{q}},\nu }^{I}$ represents the eigenvector for atom $I$ with mass ${M}_{I}$.

Then, we perform integration over the energy of the Si OM and over the physical aperture size and location in momentum space. We replace $\nu$ with Si OM as well. We then obtain an expression for the EELS phonon intensity in the DMP spectra:

2 $${I}_{{\rm{D}}{\rm{M}}{\rm{P}}}=-\frac{4\hslash }{{{a}_{0}}^{2}}{\int }_{{\omega }_{{\rm{S}}{\rm{i}}{\rm{O}}{\rm{M}}}}{\rm{d}}\omega ({\int }_{{\Delta }_{+}}-{\int }_{{\Delta }_{-}})\frac{{\rm{d}}\Omega }{{q}^{4}}\frac{{f}_{{\boldsymbol{q}},{\rm{S}}{\rm{i}}{\rm{O}}{\rm{M}}}+1}{{\omega }_{{\boldsymbol{q}},{\rm{S}}{\rm{i}}{\rm{O}}{\rm{M}}}}{|\sum _{I}\frac{1}{\sqrt{{M}_{I}}}{F}_{I}({\boldsymbol{q}}){\boldsymbol{q}}\cdot {{\boldsymbol{e}}}_{{\boldsymbol{q}},{\rm{S}}{\rm{i}}{\rm{O}}{\rm{M}}}^{I}{e}^{-i{\boldsymbol{q}}\cdot {{\boldsymbol{\tau }}}_{I}}|}^{2}\delta (\omega -{\omega }_{{\boldsymbol{q}},{\rm{S}}{\rm{i}}{\rm{O}}{\rm{M}}}).$$

The key aspect of this expression is the differential across the diametrically opposed aperture locations and the non-equilibrium phonon population. The specularity of interfaces creates an anisotropy in the non-equilibrium phonon population, which provides the contrast in the DPM map.

When compared to the on-axis, momentum-averaged mapping in Fig. 3a, the intensity of the Si OM mode is higher in the QD for the Δ_+_ region than the interlayer Si (Extended Data Fig. 9b), despite a lower Si concentration, suggesting that there is a stronger preference for phonons to have a downward momentum in the QD. The same intensity enhancement as seen in Fig. 3a is recovered when summing intensity from both Δ_+_ and Δ_–_ regions (Extended Data Fig. 9d).

### Inference of phonon MFP using reflection intensities

The reflection-induced non-equilibrium phonon population *g*′ decays as a function of distance from the interface, with the decay length being the MFP of the Si OM (Extended Data Fig. 6). A 20 nm × 15 nm mapping of the interlayer Si bounded by two QDs illustrates a gradual change in the Si OM intensity (Extended Data Fig. 7). The top of the region is bounded by an abrupt interface, whereas the bottom is bounded by a gradual interface. With the consideration that both interfaces have some degree of specularity, the data were fit with the sum of the two exponential decays arising from both the interfaces. With the fit, we obtained an MFP (*Λ*) value of 50.2 ± 19.4 nm for the Si OM, which is within an order of magnitude of accepted values, being in the range of tens of nanometres^44^, demonstrating that the MFP of phonons can be measured at nanometre resolution.

## Online content

Any methods, additional references, Nature Research reporting summaries, source data, extended data, supplementary information, acknowledgements, peer review information; details of author contributions and competing interests; and statements of data and code availability are available at 10.1038/s41586-022-04736-8.

## Supplementary information

Supplementary Information Supplementary Sections 1–6, Figs. 1–7 and references.

## Data Availability

Please contact X.Q.P. for all STEM imaging, EELS data and code, and BTE-related data.
