# Supplementary material for: Nanoscale imaging of phonon dynamics by electron microscopy
Source: Nature. 2022 Jun 8;606(7913):292–7. doi: 10.1038/s41586-022-04736-8 (PMC9177420; doi:10.1038/s41586-022-04736-8)
Supplement: Supplementary file 1 — Supplementary Sections 1–6, Figs. 1–7 and references. [file 41586_2022_4736_MOESM1_ESM.pdf]

nature portfolio

https://doi.org/10.1038/s41586-022-04736-8

Supplementary information

Nanoscale imaging of phonon dynamics by electron microscopy

In the format provided by the authors and unedited

Supplementary Information:
Nanoscale Imaging of Phonon Dynamics by Electron Microscopy

Chaitanya A. Gadre[1,6], Xingxu Yan[2,4,6], Qichen Song[3], Jie Li[1], Lei Gu[1], Huaixun Huyan[2], Toshihiro Aoki[4], Sheng-Wei Lee[5], Gang Chen[3], Ruqian Wu[1], Xiaoqing Pan[1,2,4]*

[1]Department of Physics and Astronomy, University of California Irvine, CA 92697, United States

[2]Department of Materials Science and Engineering, University of California Irvine, CA 92697, United States

[3]Department of Mechanical Engineering, Massachusetts Institute of Technology, Cambridge, MA 02139, United States

[4]Irvine Materials Research Institute, University of California Irvine, CA 92697, United States

[5]Institute of Materials Science and Engineering, National Central University, Taoyuan, 32001 Taiwan

[6]These authors contributed equally: Chaitanya A. Gadre and Xingxu Yan

*xiaoqing.pan@uci.edu

This file contains Supplementary Sections SI1–SI6, Supplementary Figs. S1–S7, and References.

SI 1: Selection rules of vibrational EELS and Raman
SI 2: Local Phonon Density of States using lattice dynamics calculations
SI 3: Effect of Strain on the Si OM
SI 4: Estimations of beam-induced phonon generation and temperature rise
SI 5: Determination of the Reflection Coefficient by Atomistic Green's Function
SI 6: Phonon population from Boltzmann transport equation

Figure S1 | Local Phonon Density of States of a SiGe nanostructure.
Figure S2 | Strain effect on Phonon Dispersion and Density of States.
Figure S3 | Phonon generation and reflection schematic.
Figure S4 | High-range EELS for average energy loss on interlayer Si.
Figure S5 | The reflection coefficients for phonon states of silicon through [001] direction.
Figure S6 | Interface specularity vs. geometry.
Figure S7 | Effect of interface geometry and specularity on phonon reflection.

SI 1: Selection rules of vibrational EELS and Raman

The differential cross section for Stokes Raman scattering writes[1],

$$\frac{d^2\sigma}{d\Omega d\omega} = \sum_\nu \frac{\omega_S^4}{c^4} \left| \boldsymbol{e}_S \cdot \overline{\overline{\boldsymbol{R}}}^\nu \cdot \boldsymbol{e}_L \right|^2 (f_\nu + 1)\delta(\omega - \omega_\nu), \tag{1}$$

where f_ν is the Bose-Einstein distribution, ω_s and ω_ν are the frequency of scattered light and phonon ν respectively, $\boldsymbol{e}_S$ and $\boldsymbol{e}_L$ are the polarization of scattered and incident light, c is the speed of light, and $\overline{\overline{\boldsymbol{R}}}^\nu$ is the Raman tensor for phonon ν, defined by

$$R_{\alpha\beta}^\nu = \sqrt{\frac{V_0 \hbar}{2\omega_j}} \sum_{\nu=1}^N \frac{\partial \chi_{\alpha\beta}^\infty}{\partial \boldsymbol{R}_i} \cdot \frac{\boldsymbol{e}_i(\nu)}{\sqrt{M_i}}, \tag{2}$$

where V_0 is the unit cell volume, $\boldsymbol{R}_i$ is the position of i th atom, N is the number of atoms in the unit cell, $\boldsymbol{e}_i(\nu)$ is the eigenvector of phonon branch ν, and $\chi_{\alpha\beta}^\infty$ is the electronic susceptibility in the limit of $\boldsymbol{q} \to 0$. This is because the momentum of phonon $\boldsymbol{q}$, transferred by the photon, is almost at the zone center.

At the heart of the expression lies the selection rule that governs Raman processes:

$$\boldsymbol{e}_S \cdot \frac{\partial \chi_{\alpha\beta}^\infty}{\partial \boldsymbol{R}_i} \cdot \frac{\boldsymbol{e}_i(\nu)}{\sqrt{M_i}} \cdot \boldsymbol{e}_L.$$

First, the potency of the Raman response is strengthened if the polarization direction of the incident light aligns with the polarization of the *i*th atom or molecule. When using unpolarized incident photons, only the projection of their polarization that aligns with that of the atoms or molecules results in a Raman excitation. Furthermore, it is necessarily the case that the initial and final states of the system must result in a change in polarizability as indicated by $\frac{\partial \chi_{\alpha\beta}^\infty}{\partial \boldsymbol{R}_i}$. Lastly, due to the

extremely steep dispersion of light relative to phonons, Raman processes can only excite nearly zone-center ($\boldsymbol{q} \rightarrow 0$) phonons.

For EELS, the corresponding scattering matrix element is the same as Eq. 1 in the methods section of the main text. The dominant factor that contributes to the vibrational EELS section rules is the product of effective charge density, $\boldsymbol{q}$, and $\boldsymbol{e}_{\boldsymbol{q},\nu}$ which, like in the Raman case, represents the polarization of the *i*th atom. However, unlike photons in the Raman process, electrons can undergo large momentum exchanges such that phonons of all momenta can be excited but only the projection of the electron momentum transfer onto the atomic displacement vector contributes to the scattered intensity. Additionally, due to the large incident momentum of the fast electron and the extremely small scattering angle, the momentum exchange in the beam direction is minimal and predominantly along the direction perpendicular to the beam direction. As a result, phonon modes that involve atomic motion along the beam direction are very weak. In most cases, an on-axis geometry is employed which is analogous to using unpolarized light in the Raman case, that allows for a wide distribution of deflections of electrons, but EELS has the added benefit of varying its momentum via probe size. EELS is highly sensitive to the effective charge density of the ionic cores[2]. To elucidate, metallic and semi metallic materials whose valence electrons can properly screen the ionic cores will greatly suppress low momentum optical modes. This only means that at a certain momentum, certain modes are suppressed. In contrast to Raman spectroscopy, all vibrational modes are active at least some momentum and polarization. Using an on-axis beam geometry ensures a maximum likelihood of exciting all planar modes.

Quantitatively speaking, the probability of exciting a phonon is about 1 in 10^3, as seen in Fig. 3a and Extended Data Fig. 3 with normalized EELS units, compared with 1 in 10^8 for Raman

scattering[3]. Therefore, the scattering efficacy of electrons, at least for on-axis geometries, is about 10^5 times higher than photons.

SI 2: Local Phonon Density of States using lattice dynamics calculations

The density of states for an abrupt and a gradual interface are calculated to see if local density of states of Si is modified by the presence of the Si/Ge QD. The local density of states is obtained from the imaginary part of Green's function, defined by $D(\omega, \boldsymbol{R}_i) = \sum_{\boldsymbol{q}\nu} u^*_{\boldsymbol{q}\nu,\alpha}(\boldsymbol{R}_i) u_{\boldsymbol{q}\nu,\alpha}(\boldsymbol{R}_i)\delta(\omega - \omega_{\boldsymbol{q}\nu})$, where $u_{\boldsymbol{q}\nu,\alpha}$ is the phonon eigenvector along α direction and $\boldsymbol{R}_i$ is the position of ith atom. We use tetrahedron smearing to sample the states with certain frequency [4]. The force constant is calculated from Stillinger-Weber (SW) potential [5] with lattice constant chosen as 5.431 Å. A 20×20×18 *k* point mesh is used to calculate density of states. Our calculation shows that, the local density of states of Si are insensitive to the interface structure (Fig. S1). With this, we affirm that the local density of states does not contribute to the enhancement of EELS intensity.

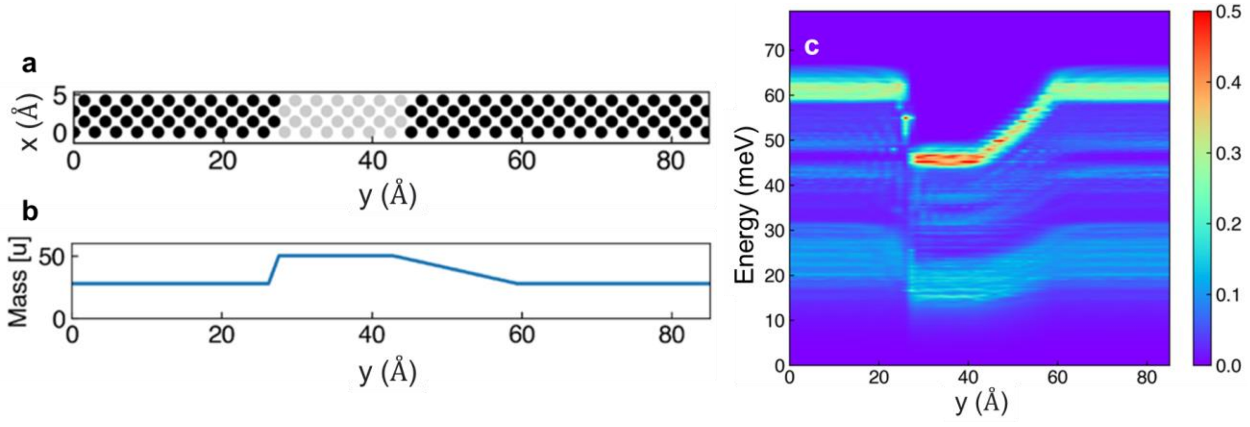

Figure S1 | Local Phonon Density of States of a SiGe nanostructure. a, Superlattice supercell with SiGe alloy structure formed by atoms representing the average mass of Si and Ge with the gray color indicating an atomic mass of $Si_{0.5}Ge_{0.5}$. The 12 layers of atoms to the right of the gray colored ones have a mass gradient that transitions from $Si_{0.5}Ge_{0.5}$ to pure Si. **b**, Mass profile of **a** along the *y*-direction. Near the abrupt Si-QD interface, the mass abruptly changes. The gradual interface is modeled by continuously changing the mass profile as a function of position. **c**, The local density of phonons for vibrational modes along the *y*-direction in the superlattice depicted in **a**.

SI 3: Effect of Strain on the Si OM

We also investigated if the strain could lead to the observed enhancement in EELS. The functional type for the pseudopotential is the Projector Augmented Wave[6] with Perdew-Burke-Ernzerhof (PBE) exchange-correlation and nonlinear core correction[7]. The energy cutoff is 36 Ry and a 4×4×4 Monkhorst-Pack mesh is used. The lattice constant at equilibrium (zero strain) is found to be 5.4662 Å by minimizing the total energy. 2×2×2 supercells with 64 atoms are used for calculating the harmonic force constant. The DFT calculation is conducted using Quantum ESPRESSO package[8] and the phonon calculation is carried out using phonopy[9] package. Through DFT calculation of phonon band and density of states of Si with different strain, we find the strain effect always modifies the phonon density of states and shifts the frequency simultaneously (Fig. S2), whilst in experiment, we only observe intensity difference of EELS but not the phonon frequency in Si (Figs. 2b and 3a). This suggests strain effect cannot explain the experimental observation.

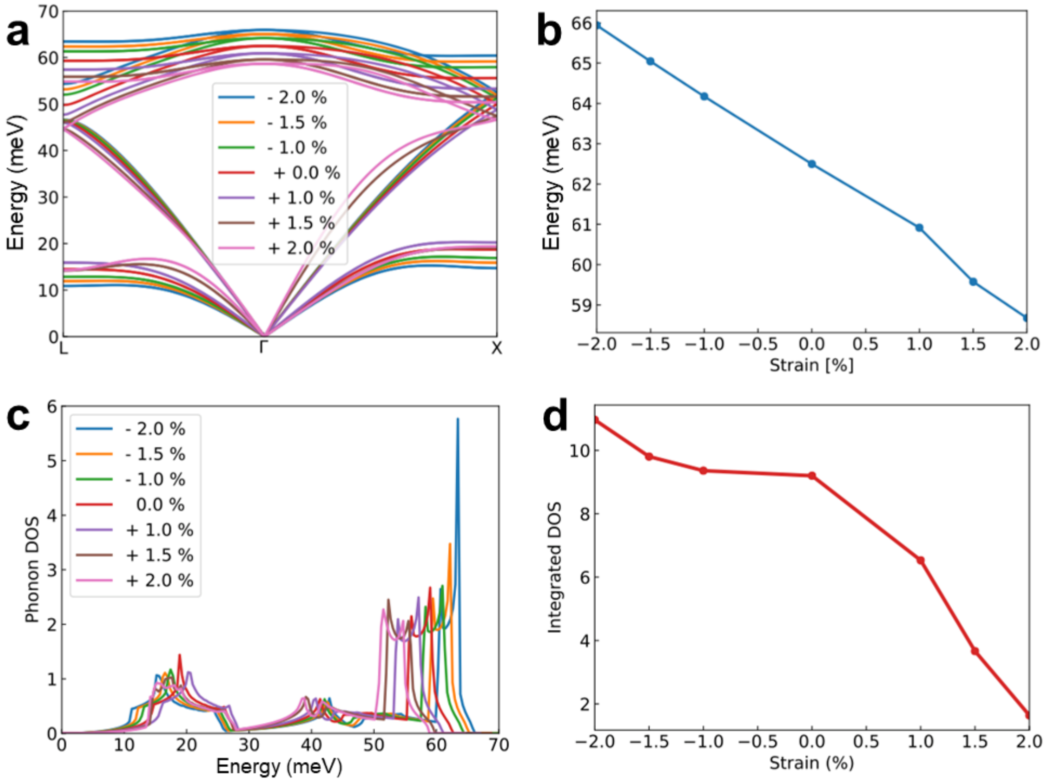

Figure S2 | Strain effect on Phonon Dispersion and Density of States. a, Phonon dispersion for silicon under different strains. **b**, The energy of longitudinal phonon at Γ point as a function of strain. **c**, Phonon density states from DFT calculations for different strains. The percentage marks the relative change in lattice constant compared to equilibrium state. **d**, The integrated phonon density of states from 55 meV to 65 meV. As the lattice constant enlarges, the integrated DOS in the energy window from 55 meV to 65 meV decreases due to the red shift and spreading of optical phonons.

SI 4: Estimations of beam-induced phonon generation and temperature rise

A schematic depicting the generation of phonons by the electron beam is given in Fig. S3. In the following, we will estimate how many phonons are generated by the electron beam per unit time. The electron beam current through the sample is measured to be 3 pA meaning that there are 0.019 electrons travelling through sampler per ns. If we consider the beam as a stream of consecutive single electrons, the average time interval between electrons transmitting through sample is 53 ns. By examining the EELS intensity, $I(\Delta E)$, we estimate the average energy loss per electron: $<\Delta E> = \int(\Delta E)I(\Delta E)\mathrm{d}\Delta E/\int I(\Delta E)\mathrm{d}\Delta E = 34.6$ eV, where ΔE denotes the energy loss of the fast electron (Fig. S4). Although the energy loss is in various forms, for example plasmons, eventually the energy loss all become heat energy to excite phonons with various decay times. From the density of states computed using first-principles calculations, we find the average phonon energy $<E_{ph}>=\int\hbar\omega D(\omega)\mathrm{d}\omega/\int D(\omega)\mathrm{d}\omega = 40.4$ meV. Then, the number of Si OM phonons per electron is given by $\frac{\langle \Delta E\rangle}{\langle E_{ph}\rangle}\int_{\omega_1}^{\omega_2} D(\omega)\mathrm{d}\omega \Big/ \int D(\omega)\mathrm{d}\omega = 291$ where $\hbar\omega_1$=55 meV to $\hbar\omega_2$=65 meV. This calculation represents the upper limit for the number of phonons that survive until the next electron arrives. With 291 Si OM phonons per electron, we can calculate the beam-induced non-equilibrium phonon generation rate $\dot{n} = 5.43\times10^9$ s^{-1} for Si OM phonons. In addition, from the first-principles calculation, we find that the volume-specific heat capacity for optical phonons is $C_{\mathrm{p,OM}} = 4.53\times10^5$ J/m^3/K. If further assuming the energy of those optical phonons are uniformly confined within the range of mean free path, *i.e.*, with the volume of a cylinder of $\pi\times(10\text{ nm})^2\times50$ nm where 50 nm is the thickness of the slab, the effective temperature rise due to optical phonons is estimated to be

0.36 K, which is negligible compared to room temperature 300 K. Thus, the equilibrium part of the phonon occupation number is well described by the Bose-Einstein distribution at 300 K.

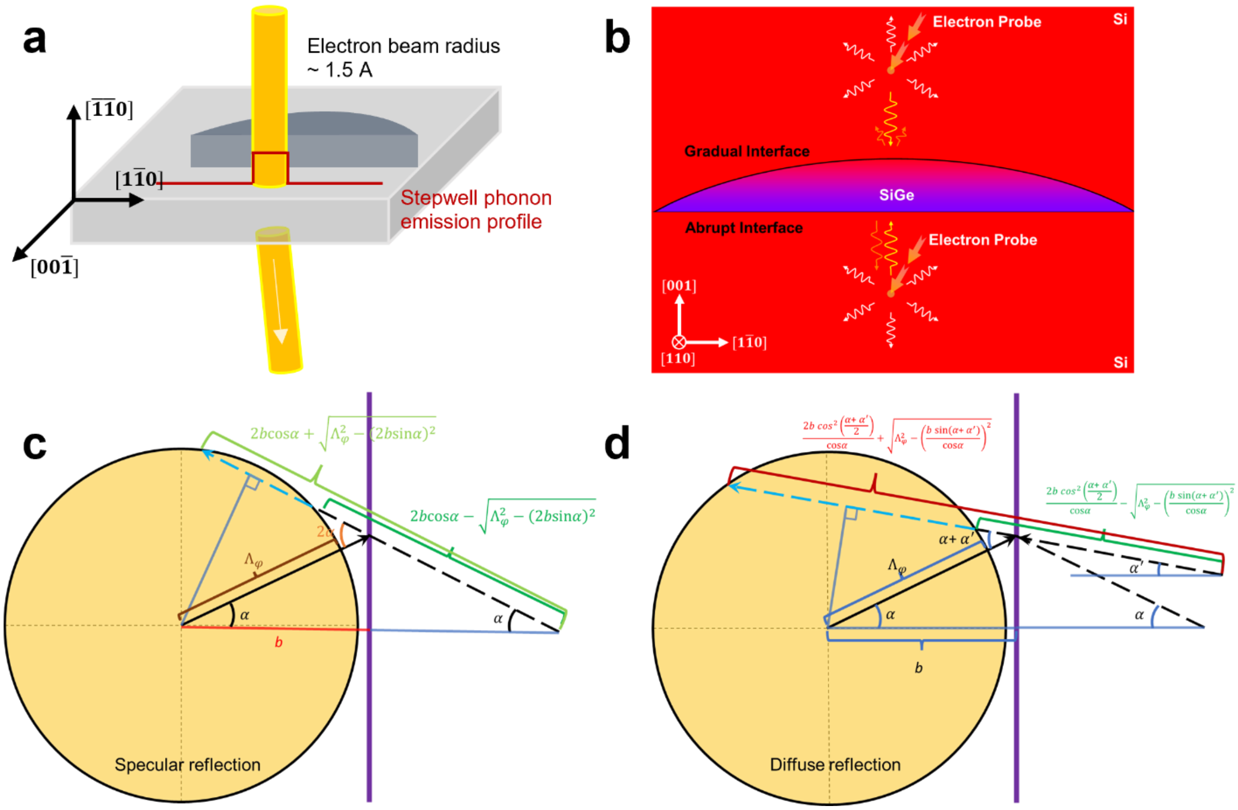

Figure S3 | Phonon generation and reflection schematic. a, Schematic for the electron beam as a phonon emission source in EELS. The crystal directions are noted to the left. **b**, Two-dimensional geometry from top view with crystal directions included in the bottom left. The dark region indicates the quantum dot. The beam is indicated by the orange circle and its area is exaggerated for visual convenience. The arc measured is found be about 36.5° with the radius around 138.8 nm. The mean free path of Si OM phonons is chosen as 10 nm with a group velocity of 1000 m/s. **c-d**, Specular and diffuse reflection geometries, respectively. The yellow circle denotes the area over which the electron beam can sense phonons. The vertical purple lines denote the interfaces. The cyan dashed arrows in both **c** and **d** denote the area over which the reflected phonons are measured by the electron beam. For specular reflection, the reflected angle is equal to the angle of incidence and for diffuse reflection, the reflected angles are spread over angles $-\pi/2 < \alpha' < \pi/2$. The same crystal directions as in **b** apply.

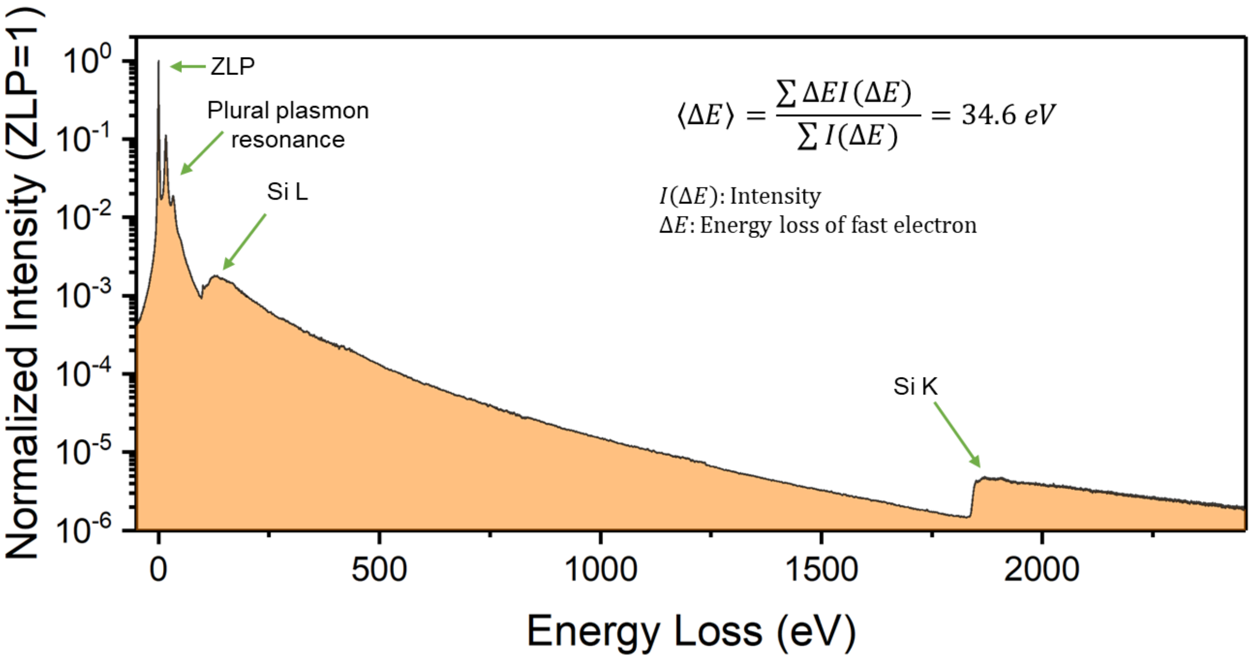

Figure S4 | High-range EELS for average energy loss on interlayer Si. An EEL spectrum contains a probability-like distribution of the energy loss of single electron. This high-range spectrum shows plural plasmon resonance, Si *L* edge, and Si *K* edge that, assuming nonradiative losses in Si, eventually decay into phonons. Weighting by Si DOS, there are 291 Si OM phonons generated per fast electron.

SI 5: Determination of the Reflection Coefficient by Atomistic Green's Function

To obtain the reflection coefficients for abrupt and gradual interfaces, we followed Khomyakov's mothed of using Green's function to calculate reflection matrix[10]. We first constructed a silicon-quantum dot interface structure, where the leads are semi-infinite silicon and quantum dot along [001] direction, respectively and the central device is the silicon/quantum dot interface. The mass of the quantum dot lead region is 33.1u, which corresponds to a virtual atom of mass equal to 0.89m_{Si} + 0.11m_{Ge}. The retarded Green's function for the device is written as, $G^{ret}(\omega, \boldsymbol{k}_{\parallel}) = \left(\omega^2 I - D_C(\boldsymbol{k}_{\parallel}) - \Sigma_L(\omega, \boldsymbol{k}_{\parallel}) - \Sigma_R(\omega, \boldsymbol{k}_{\parallel})\right)^{-1}$, where $D_C(\boldsymbol{k}_{\parallel})$ is the dynamical matrix of device with transverse momentum $\boldsymbol{k}_{\parallel}$, which is parallel to the interface of the superlattices. The self-energy of left and right lead is $\Sigma_{L/R}(\omega, \boldsymbol{k}_{\parallel}) = V_{L/R} g_{L/R}^{ret} V_{L/R}^{\dagger}$ and $g_{L/R}^{ret}$ is the surface Green's function, which is calculated using the decimation technique and $V_{L/R}$ is the submatrix of dynamical matrix describing the coupling between the lead and the central device. The reflection matrix is $r_{LL} = \frac{2i\omega}{a_L}\sqrt{V_L^{ret}}[U_L^{ret}]^{-1}(G_L^{ret} - Q_L^{-1})[U_L^{adv\dagger}]^{-1}\sqrt{V_L^{adv}}$, where $V_L^{adv/ret}$ is the velocity matrix, $U_L^{adv/ret}$ is the matrix of eigenvectors and a_L is the lattice constant in transport direction. The reflection coefficient for i th phonon mode in left lead is $R^L{}_i = \sum_j (|r_{LL}|^2)_{ij}$. We use Stillinger-Weber (SW) potential[5] to calculate the dynamical matrix, which generally leads to slightly overestimated phonon frequencies of silicon. Fig. S5 shows that there is a small difference between the abrupt and gradual interfaces.

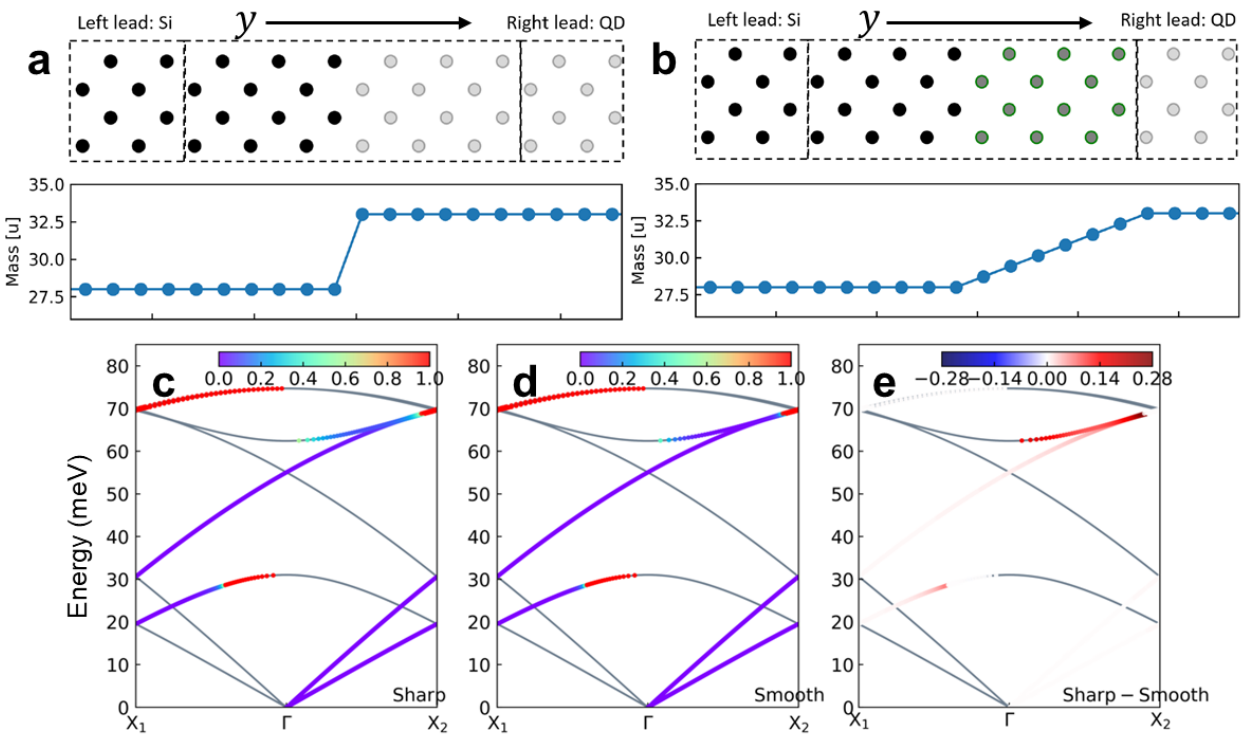

Figure S5 | The reflection coefficients for phonon states of silicon through [001] direction. a, **b**, Atomic structure (upper panel) and corresponding mass profile (lower panel) of abrupt Si-QD interface **a** and gradual Si-QD interface **b**. In the atomic structures, the darker the atom's color is, the lighter the atomic mass is. For an atomic layer with the same z position, the mass is the same. Periodic boundary conditions in transverse directions are used. **c-e**, Reflection coefficients of abrupt Si-QD interface **c**, gradual Si-QD interface **d** and their difference **e** along [001] direction in reciprocal space. The transport direction normal to the interface is along z direction. The high symmetry point X_1 refers to $(0,0,-1)\frac{\pi}{a}$ and point X_2 refers to $(0,0,1)\frac{\pi}{a}$ in the first Brillouin zone of the 4-atom unit cell where x direction is [001] direction. The modal reflection coefficients are calculated from atomistic Green's function.

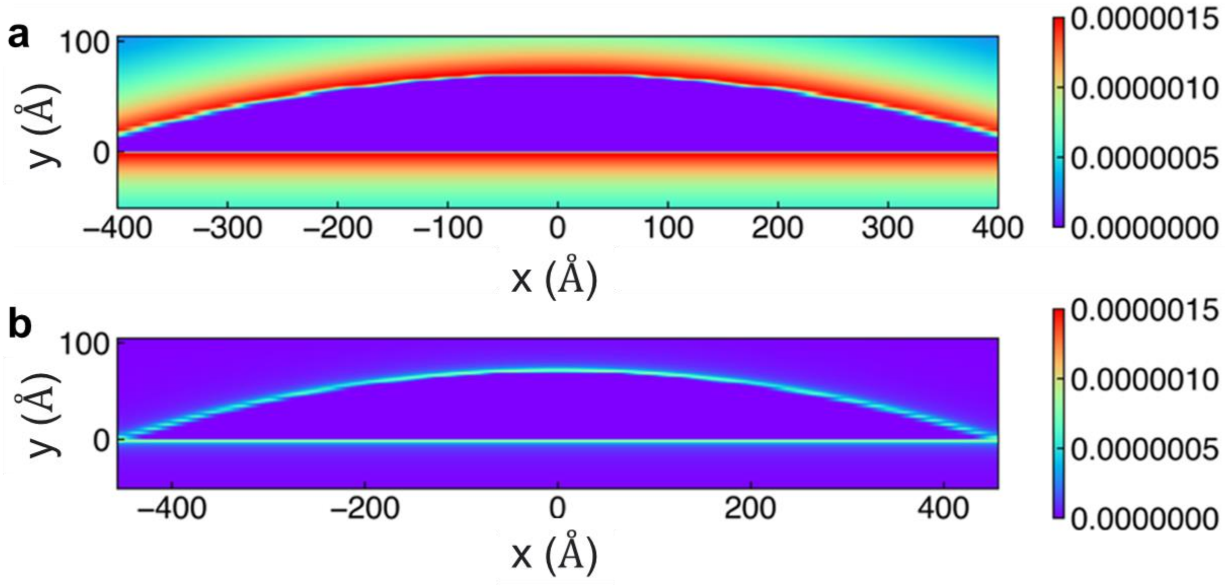

Figure S6 | Interface specularity vs. geometry. a, Spatially integrated distribution function of specularly reflected phonons with directions of incoming phonon velocity normal to and away from interface. Here, we assume $S = R = 1$ for both abrupt and gradual interface. Since SR is identical for abrupt and gradual interfaces, the reflected phonon follows a similar decay trend. In practice, the SR are different for two interfaces, causing asymmetry in the phonon distribution function. **b**, Diffusively reflected phonon distribution function shows strong localized feature near the surface of quantum dot, assuming $S = 0$ for both interfaces.

SI 6: Phonon population from Boltzmann transport equation

To properly describe the nonequilibrium phonon population near an interface, the phonon Boltzmann transport equation (BTE) with source within the relaxation time approximation writes[11],

$$\frac{\partial f_{q\nu}}{\partial t} + v_{\boldsymbol{q}\nu} \cdot \nabla f_{\boldsymbol{q}\nu} = -\frac{f_{\boldsymbol{q}\nu} - f_{\boldsymbol{q}\nu,0}}{\tau_{\boldsymbol{q}\nu}} + \dot{n}_A, \tag{3}$$

where $f_{q\nu}$ is the phonon distribution function, $v_{\boldsymbol{q}\nu}$ is the group velocity, $f_{\boldsymbol{q}\nu,0}$ is the equilibrium phonon population i.e., the Bose-Einstein distribution, $\tau_{\boldsymbol{q}\nu}$ is the phonon lifetime and $\dot{n}_A$ is the phonon generation rate per unit time per unit area due to the energy transferred from electron beam to sample. $\dot{n}_A = \dot{n} / A_e$ with A_e as the area of electron beam (πa^2), where a is the radius of electron beam (a = 0.075 nm for 33 mrad convergence semi-angle condition). Define $g_{\boldsymbol{q}\nu}$ as the nonequilibrium part of the phonon population as: $g_{\boldsymbol{q}\nu} = f_{\boldsymbol{q}\nu} - f_{\boldsymbol{q}\nu,0}$.

As discussed in above section, the temperature rise is negligible thus $f_{\boldsymbol{q}\nu,0}$ is approximated to be a constant for all modes. Additionally, since the electrons constantly hit the sample slab, we treat this process as quasi-steady state. Thus, the time-derivative of the phonon population ($\partial f_{\boldsymbol{q}\nu}/\partial t$) is dropped. As a result, Eq. 3 becomes,

$$v_{\boldsymbol{q}\nu} \cdot \nabla g_{\boldsymbol{q}\nu} = -\frac{g_{q\nu}}{\tau_{\boldsymbol{q}\nu}} + \dot{n}_A. \tag{4}$$

We chose cylindrical coordinate for mathematical convenience because of the cylindrical nature of the electron beam transmitting through the slab with the z axis along the beam axis labeled as $[\bar{1}\bar{1}0]$ (Fig. S3). We set the origin to be the center of the beam and we then solve the Boltzmann transport equation (BTE) by Eq. 4.

$$\left(v_r\hat{\boldsymbol{r}} + v_\theta\hat{\boldsymbol{\theta}}\right)\cdot\boldsymbol{\nabla}g(r,\theta,v_r,v_\theta) = -\frac{g(r,\theta,v_r,v_\theta)}{\tau} + \dot{n}_A(r,\theta), \tag{5}$$

where we drop the superscript and subscript for clarity. In relation to the QD structure in Fig. 1a, a denotes the diameter of the electron beam and r denotes the distance from the beam. In the derivation, θ represents the angular spread of the phonons generated by the electron beam. The phonon source is represented by a step function, $\dot{n}(r,\theta) = \Theta(-r)$. The radial and tangential component of velocity are $v_r = v_y\sin\theta + v_x\cos\theta$ and the $v_\theta = v_y\cos\theta - v_x\sin\theta$. When the phonon source is located far from the interface, the boundary conditions of phonon BTE are $g(0,\theta,|v_r|,v_\theta) = g(0,\theta,-|v_r|,v_\theta)$, g is continuous at $r = a$, and $g(v\tau,\theta,-|v_r|,v_\theta) = 0$, where $v = \sqrt{v_x^2 + v_y^2}$. Thus, the solution[12] is,

$$g(r) = \begin{cases} \dot{n}_A\tau\left(1 - e^{-\frac{v_r r - \sqrt{a^2v^2 - r^2v_\theta^2}}{v^2\tau}}\right), r < a \\ 2\dot{n}_A\tau e^{-\frac{v_r r}{v^2\tau}}\sinh\left(\frac{\sqrt{a^2v^2 - v_\theta^2 r^2}}{v^2\tau}\right), r \geq a \end{cases}, \tag{6}$$

Eq. 6 describes a quasi-1D trajectory along (v_r, v_θ) as depicted in Fig. S7.

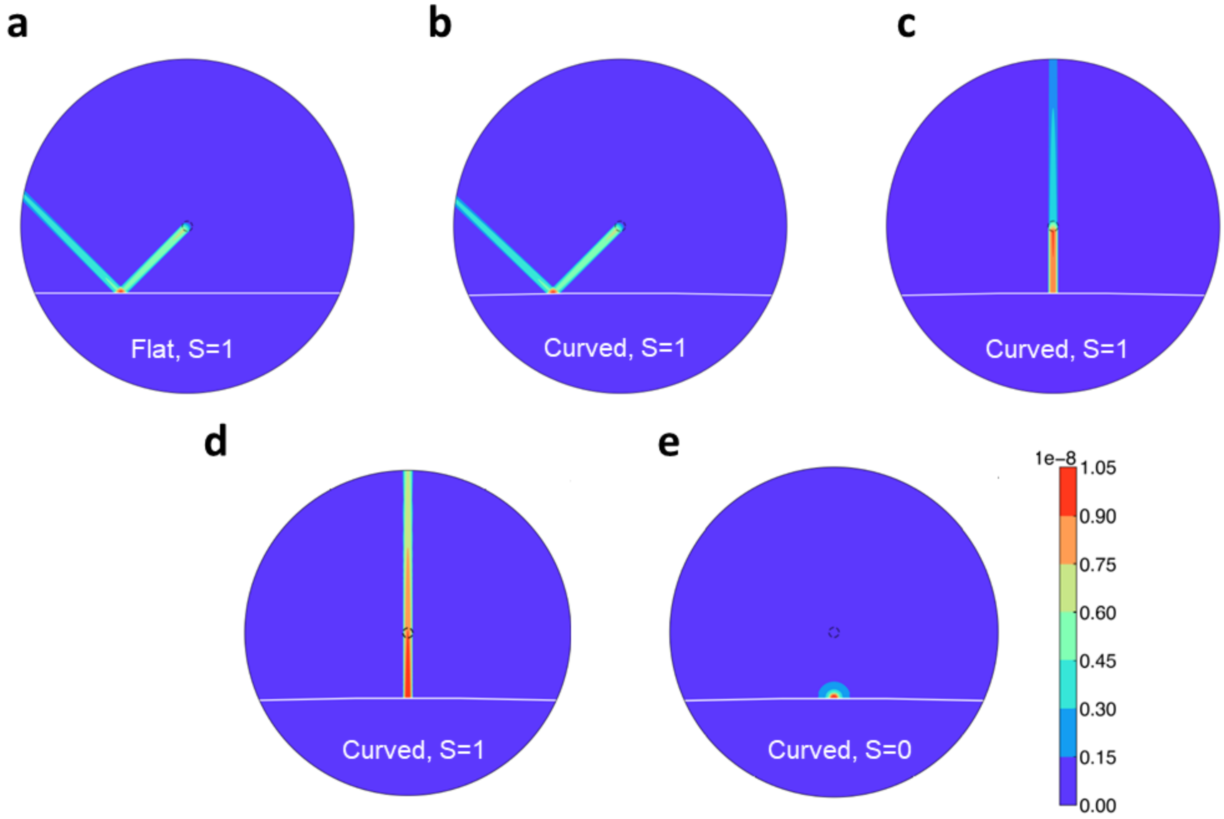

Figure S7 | Effect of interface geometry and specularity on phonon reflection. a-c, The non-equilibrium phonon distribution function g for a phonon mode along certain polar angle that consists of the incoming and corresponding fully specularly reflected phonons (S=1, R=1) near a flat **a** and curved **b**, interface with source phonon moving with along θ = -135° and a curved interface **c**, with source phonon moving along θ = -90°, respectively. We find that only normal incident phonons have significant enhancement in the nonequilibrium phonon population. **d**, **e**, The fully specularly reflected (S=1, R=1) and diffusively scattered (S=0, R=1) nonequilibrium phonon distribution $g'(r', v_r', v_\theta')$ and $g''(r)$ respectively, when the incoming phonon at normal to the interface. Only reflected phonon population are plotted for **d** and **e**. From **e**, we find the diffusively scattered part is highly localized near the interface such that we can neglect the enhancement of phonon population from the contribution of diffusively scattered phonons. The small, dashed circles in the center of **a-e** indicate the location of the phonon emission source. The color bar represents the phonon population with arbitrary units.

To obtain a 2D distributed population, we begin with a polar integration of Eq. 6 while also integrating over the angular distribution α of the quasi-1D trajectory:

$$g(r) = \frac{1}{2\pi}\int_{\alpha_1}^{\alpha_2} \dot{n}_A d\alpha \int_{r_1}^{r_2} r dr \int_0^{2\pi} d\theta\, g(r,\theta,v_r,v_\theta). \quad (7)$$

For $r > a$, we can approximate $g(r,\theta,v_r,v_\theta)$ and simplify the integral:

$$g(r) = \frac{1}{2\pi}\int_{\alpha_1}^{\alpha_2} \dot{n}_A d\alpha \int_{r_1}^{r_2} r dr \int_{-\frac{a}{r}}^{\frac{a}{r}} d\theta\, 2\tau e^{-\frac{r}{\Lambda}} \frac{a}{\Lambda}. \quad (8)$$

The general result without inclusion of specific reflection geometries is:

$$g(r) = \frac{2}{\pi^2}\dot{n}\tau \frac{1}{\Lambda}\int_{\alpha_1}^{\alpha_2} \left(e^{-\frac{r_1}{\Lambda}} - e^{-\frac{r_2}{\Lambda}}\right) d\alpha. \quad (9)$$

Here, r_1 and r_2 are the bounds within which the population distribution is sensed by the electron beam, indicated by blue dashed arrows in Fig. S3. This radius of this area is known as the phonon coherence length Λ_φ. We argue that the although the electron beam is extremely small in radius, the phonons generated are populated in a larger area due to the planewave nature of phonons. The phonons that are excited by the fast electron and keep their memory of origin, shall be within the phonon coherence length[13] from the beam center. For inelastic scattering dominated phonons, the coherence length is proportional to the inelastic phonon mean free path, $\Lambda_\varphi = \Lambda_{in}/\sqrt{3}$, where Λ_{in} is the phonon mean free path. In the Si side, the dominate scattering mechanisms are inelastic phonon-phonon interactions such that $\Lambda = \Lambda_{in}$. Thus, we have the cutoff radius $\Lambda_\varphi = \Lambda/\sqrt{3}$. Since the beam radius is typically much smaller than the phonon mean free path Λ, we can obtain an approximated form of Eq. 9.

The expressions for beam generated, specularly reflected, and diffusely reflected phonons, respectively, are given below. For brevity, only solutions where $b > \Lambda_\varphi$ are given.

$$g_0 = \frac{4}{\pi}\dot{n}\tau\left(1 - e^{-\frac{\Lambda_\varphi}{\Lambda}}\right), \quad (10)$$

$$g' = \frac{8}{\pi^2}\dot{n}\tau \int_0^{\sin^{-1}\left(\frac{\Lambda\varphi}{2b}\right)} e^{-\frac{2b\cos\alpha}{\Lambda}} \sinh\frac{\sqrt{\Lambda_\varphi^2-(2b\sin\alpha)^2}}{\Lambda} d\alpha, \quad (11)$$

and

$$g'' = \frac{4}{\pi^3}\dot{n}\tau \int_0^{\frac{\pi}{2}} d\alpha \int_{-\sin^{-1}\left(\frac{\Lambda\varphi\cos\alpha}{b}\right)-\alpha}^{\sin^{-1}\left(\frac{\Lambda\varphi\cos\alpha}{b}\right)-\alpha} e^{-\frac{2b\cos^2\left(\frac{\alpha+\alpha'}{2}\right)}{\Lambda\cos\alpha}} \sinh\frac{\sqrt{\Lambda_\varphi^2-\left(\frac{b\sin(\alpha+\alpha')}{\cos\alpha}\right)^2}}{\Lambda} d\alpha' \ . \quad (12)$$

Near an interface, the total phonon population is:

$$f = f_0 + g_0 + S\, R_s\, g' + (1-S)R_d\, g'', \quad (13)$$

where g_0 is nonequilibrium phonon population directly generated by incident electron beam, g' is the specular reflection of nonequilibrium phonon from the interface, and g'' is the diffuse reflection of nonequilibrium phonon from the interface. Here S is the specularity parameter at the interface ($0 \leqslant S \leqslant 1$), R_s and R_d are the specular and diffuse reflectance of the interface ($0 \leqslant R \leqslant 1$), respectively.

We first consider specular reflection with oblique and normal incidence and find only normal incidence towards interface causes enhancement in the nonequilibrium phonon population, shown in Figs. S7a–S7c. Then, we consider the case of reflected nonequilibrium phonon population from diffuse reflection, $g''=\int g_d(\alpha)\, d\alpha / 2\pi$, where $g_d(\alpha)$ is the diffusely reflected phonon population with α being the angle for diffusely reflected phonons. As shown in Figs. S7d and S7e, we find the diffuse reflection does not contribute to the phonon population enhancement except extremely close to interface on the order of beam size, which is unresolvable in our experiment due to the larger step size. In conclusion, only specular reflection of normal incident phonons causes enhancement in nonequilibrium phonon population. In Extended Data Fig. 6, we have shown the nonequilibrium phonon population only considering normal incidence.

The expressions for specular and diffuse reflection at normal incidence are:

$$g' = 16\dot{n}\tau a^2 e^{-\frac{2b}{\Lambda}} \sinh\frac{\Lambda_\varphi}{\Lambda}, \tag{14}$$

and

$$g'' = \frac{4}{\pi^3}\dot{n}\tau \int_{-\sin^{-1}\left(\frac{\Lambda_\varphi}{b}\right)}^{\sin^{-1}\left(\frac{\Lambda_\varphi}{b}\right)} e^{-\frac{2b\cos^2\left(\frac{\alpha'}{2}\right)}{\Lambda}} \sinh\frac{\sqrt{\Lambda_\varphi^2-(b\sin(\alpha'))^2}}{\Lambda} d\alpha' \ . \tag{15}$$

To compare the enhancement ratio (Δ_{sd}) with experiment, we use the emission probability f + 1:

$$\Delta_{\mathrm{sd}} = \frac{1+f_0+g_0+S_a R_s g' + (1-S_a) R_d g''}{1+f_0+g_0+S_g R_s g' + (1-S_g) R_d g''} - 1. \tag{16}$$

Here, S_a and S_g are the specularity parameters of the abrupt and gradual interfaces, respectively. We also assume maximum secularity S_a=1, R_s=1 for the abrupt interface and we set S_g=0, R_d = 0 for the gradual interface to obtain a maximum upper bound the estimate:

$$\Delta_{\mathrm{sd}} = \frac{1+f_0+g_0+g'}{1+f_0+g_0} - 1. \tag{17}$$

Under these conditions and the values discussed in the section Estimations of beam-induced phonon generation and temperature rise, we estimate the enhancement ratio to be 17.5% This estimation is within an order of magnitude with our experimental result of 15.9% and we note that the total phonon occupation is only a part of the full scattering cross section expression and that other terms likely also influence the true quantitative value. The total phonon occupation, however, is the dominant factor in the EELS intensity enhancement.

Considering the exponential decay feature of expression in Eq. 14 from an interface with distance b, the reflected phonon population can be fit by,

$$f + 1 = A\, e^{-\frac{2b}{\Lambda}} + C. \tag{18}$$

This result is used to estimate the MFP of Si OM (Extended Data Fig. 7). We want to emphasize that although we assume the same mean free path for all optical phonons as well as same lifetime while those properties are actually momentum-dependent, our model based on BTE qualitatively rather than quantitively captures the physics of interface scattering. BTE simulation was performed by our custom python code.

References:

1. Ceriotti, M., Pietrucci, F. & Bernasconi, M. Ab initio study of the vibrational properties of crystalline TeO2: The α, β, and γ phases. *Phys. Rev. B - Condens. Matter Mater. Phys.* **73**, 104304 (2006).

2. Senga, R. *et al.* Position and momentum mapping of vibrations in graphene nanostructures. *Nature* **573**, 247–250 (2019).

3. Jones, R. R., Hooper, D. C., Zhang, L., Wolverson, D. & Valev, V. K. Raman Techniques: Fundamentals and Frontiers. *Nanoscale Res. Lett.* **14**, 231 (2019).

4. Lambin, P. & Vigneron, J. P. Computation of crystal Green's functions in the complex-energy plane with the use of the analytical tetrahedron method. *Phys. Rev. B* **29**, 3430–3437 (1984).

5. Stillinger, F. H. & Weber, T. A. Computer simulation of local order in condensed phases of silicon. *Phys. Rev. B* **31**, 5262–5271 (1985).

6. Blöchl, P. E. Projector augmented-wave method. *Phys. Rev. B* **50**, 17953–17979 (1994).

7. Perdew, J. P., Burke, K. & Ernzerhof, M. Generalized gradient approximation made simple. *Phys. Rev. Lett.* **77**, 3865–3868 (1996).

8. Giannozzi, P. *et al.* QUANTUM ESPRESSO: A modular and open-source software project for quantum simulations of materials. *J. Phys. Condens. Matter* **21**, 395502 (2009).

9. Togo, A. & Tanaka, I. First principles phonon calculations in materials science. *Scr. Mater.* **108**, 1–5 (2015).

10. Khomyakov, P. A., Brocks, G., Karpan, V., Zwierzycki, M. & Kelly, P. J. Conductance calculations for quantum wires and interfaces: Mode matching and Green's functions. *Phys. Rev. B - Condens. Matter Mater. Phys.* **72**, 035450 (2005).

11. Chen, G. Nanoscale energy transport and conversion: a parallel treatment of electrons, molecules, phonons, and photons. *Oxford Univ. Press* (2005) doi:10.5860/choice.43-2818.

12. Dingle, R. B. Electrical Conductivity of Thin Wires. *Proc. R. Soc. London* **12**, 103–109 (1950).

13. Chen, G. Non-Fourier phonon heat conduction at the microscale and nanoscale. *Nat. Rev. Phys.* **3**, 555–569 (2021).
